# A biomaterial with a channel-like pore architecture induces endochondral healing of bone defects

**DOI:** 10.1038/s41467-018-06504-7

**Published:** 2018-10-25

**Authors:** A. Petersen, A. Princ, G. Korus, A. Ellinghaus, H. Leemhuis, A. Herrera, A. Klaumünzer, S. Schreivogel, A. Woloszyk, K. Schmidt-Bleek, S. Geissler, I. Heschel, G. N. Duda

**Affiliations:** 1 Julius Wolff Institute, Charité—Universitätsmedizin Berlin, Augustenburger Platz 1, 13353 Berlin, Germany; 2 Berlin-Brandenburg Center for Regenerative Therapies, Charité—Universitätsmedizin Berlin, Augustenburger Platz 1, 13353 Berlin, Germany; 3 Matricel GmbH, Kaiserstrasse 100, 52134 Herzogenrath, Germany; 4 Berlin-Brandenburg School for Regenerative Therapies, Charité—Universitätsmedizin Berlin, Augustenburger Platz 1, 13353 Berlin, Germany; 5 Department of Orthopaedic Surgery, University of Texas Health Science Center San Antonio, 7703 Floyd Curl Dr, 78229 San Antonio, TX, USA

## Abstract

Biomaterials developed to treat bone defects have classically focused on bone healing via direct, intramembranous ossification. In contrast, most bones in our body develop from a cartilage template via a second pathway called endochondral ossification. The unsolved clinical challenge to regenerate large bone defects has brought endochondral ossification into discussion as an alternative approach for bone healing. However, a biomaterial strategy for the regeneration of large bone defects via endochondral ossification is missing. Here we report on a biomaterial with a channel-like pore architecture to control cell recruitment and tissue patterning in the early phase of healing. In consequence of extracellular matrix alignment, CD146+ progenitor cell accumulation and restrained vascularization, a highly organized endochondral ossification process is induced in rats. Our findings demonstrate that a pure biomaterial approach has the potential to recapitulate a developmental bone growth process for bone healing. This might motivate future strategies for biomaterial-based tissue regeneration.

## Abstract (web summary)

A bioengineering approach to enhance the regeneration of large bone defects is lacking. Here, the authors show that a biomaterial scaffold with a channel-like pore architecture enables organized endochondral ossification through directional cell recruitment and extracellular matrix alignment.

## Introduction

Endochondral ossification (EO) is the process of bone formation through the replacement of a cartilage anlage. All long bones in the mammalian skeleton develop through this process during the fetal stage^1^. Later, during childhood and adolescence, EO is responsible for the lengthening of the long bones at the growth plate. Current biomaterial strategies for bone defect healing primarily focus on approaches that provide osteoconductive and osteoinductive environments^2^, mainly stimulating intramembranous ossification. Clinically, however, only few fractures with direct bone contact and stable fixation heal via intramembranous ossification, but the majority of successful bone regeneration cases are proceeding through the endochondral route^3^. In this context, stimulating EO to regenerate bone has gained remarkable attention over the last years^1,4–7^ and was further strengthened through experimental evidence in vivo^8–10^. It was shown that the endochondral pathway can be supported by biomaterials when they were used for the delivery of progenitor cells, either undifferentiated^11,12^ or pre-differentiated^8–10,13^, growth factors^14,15^, or a combination of both^16–18^. Biomaterial environments, engineered to locally provide growth stimulus signals to transplanted cells, were able to support EO^19^. However, until today no purely biomaterial-based solution exists that induces EO for the regeneration of critical-size defects in long bones, while these clinical situations still represent a severe challenge.

Here we report on a unique cell- and growth factor-free approach to induce EO in large bone defects solely via biomaterial architecture. EO is linked to the presence of osteochondral progenitor cells that, e.g., reside in the bone marrow niche^20^. The recruitment of these cells into a biomaterial after in vivo implantation represents an appealing alternative to cell or tissue implantation regarding efficacy, safety, and treatment costs^21,22^. Alternative to the use of chemoattractants, our approach to provide a physical guiding structure could represent a simple, yet effective way to enhanced progenitor cell recruitment supporting the bone’s inherent capability to heal. Limited vascularization is regarded a general obstacle for biomaterial-based in vitro and in vivo tissue engineering solutions^23,24^, as vascularization might be restricted depending on the specific pore diameter and architecture^25^. However, the endochondral route of bone healing addressed here is less dependent on an initial vascular supply than strategies focusing on intramembranous ossification. This is indicated by the fact that endochondral bone development starts from avascular cartilage anlagen that are extensively vascularized only during the subsequent transition to bone^26^. Despite the presence of multiple local progenitor cell sources (marrow, periosteum, and muscle)^27^, critical-sized bone defects are severely limited in their healing capacity. Here we speculated that an unfavorable self-patterning of the extracellular matrix (ECM) limits the bone’s capacity to heal large defects by hindering cell recruitment and subsequent tissue maturation cascades, and that this could be overcome by the use of a specifically architectured biomaterial. There is growing evidence that the structure of the ECM has an important role in tissue regeneration^28,29^, and clear evidence for a coupling between extracellular structure and tissue differentiation can be found in organ development and morphogenesis^30^. First evidence exists that collagen fiber orientation can be controlled by biomaterial architecture to guide tissue mineralization^31,32^. However, more in-depth investigations are needed to employ this principle for bone tissue regeneration.

In this study, we were able to show that a biomaterial, engineered to provide mechanical properties similar to the natural ECM together with a unique channel-like macroporous architecture, enabled a structurally guided EO process across the bone defect. This was achieved by a consistent, material-controlled re-alignment of collagen fibers across the bone defect associated with enhanced progenitor cell recruitment. The biomaterial was characterized using human stromal cells and revealed its functionality in rats. Taken together, we report a purely material-based approach to induce in situ tissue regeneration via EO in large bone defects. In light of translational applications such an approach is highly desirable, as it omits the need for growth factor or cell delivery^33^.

## Results

### Characterizing collagen fiber emergence in bone defects

**Fig. 1:**
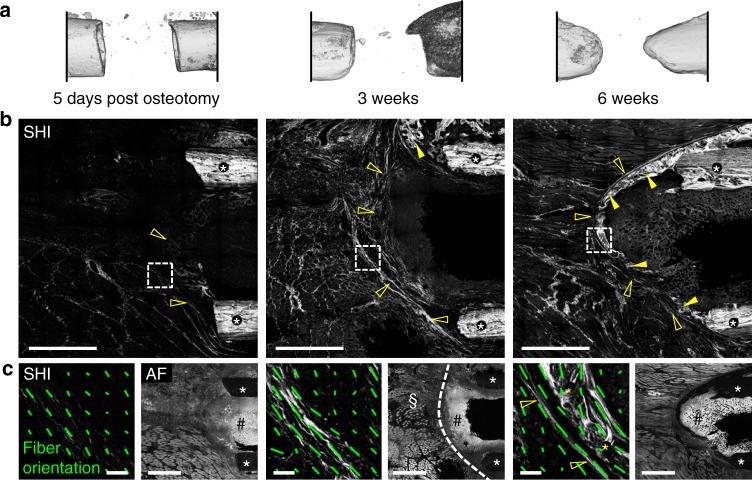
Distinct collagen fiber patterns forming in bone defect guide tissue mineralization. **a** 3D µ-CT reconstruction of the 5 mm rat femoral bone defect at different time points showing progression of tissue mineralization toward the formation of a bone-like shell closing the marrow cavity. **b** Second harmonic imaging showing the emergence of a fibrillar collagen matrix at the end of the bone (open arrowheads) preceding tissue mineralization (full arrowheads). White asterisks indicate the bone cortices. **c** Orientation analysis revealed that distinct patterns of collagen fibers develop already in the first days post osteotomy and mature towards a dense, dome-like structure until 3 weeks post-op (detailed SHI recordings at regions indicated by dashed-line squares in **b**). Green lines indicate collagen fiber anisotropy *A*_C_ (length of line) and direction of primary fiber orientation *Φ*_C_ (angle) within the local ROIs. The formation of mineralized tissue (6 weeks, post-op, yellow asterisks) is guided by aligned collagen fibers toward the formation of the marrow-closing bone shell. Autofluorescence imaging (AF) indicates the separation of the bone marrow and the periosteum (hash symbol) from the defect region (paragraph symbol) by the deposited collagen fiber structure (dashed white line, 3 weeks post-op). Scale bars for **b** 1 mm; **c** SHI-images 50 µm, AF images 1 mm

To gain information about the structural organization of the ECM, we first characterized the appearance of collagen fibers in critical-size segmental bone defects in rats via second harmonic imaging (SHI) in the absence of any biomaterial. Five days after creating the defect via an osteotomy, micro-computed tomography (µ-CT) showed only minor tissue mineralization, limited to the surface of the bone cortices (Fig. 1a). At the same time, first patterns of collagen fibers had emerged in the periosteal and endosteal region that tended to cross the marrow cavity (Fig. 1b, c, SHI). Until 3 weeks post osteotomy (post-op), a dense layer of collagen fibers had formed around the ends of the bones in a dome-like shape. Tissue mineralization was guided along the collagen fibers forming a shell-like structured callus encasing the marrow cavity until 6 weeks post-op (Fig. 1a, b). Although the formation of such a bone shell has been considered before as failure of bone healing in large defects^34^, the pre-patterning function of collagen fibers in this process was not reported so far. With the maturation of collagen fibers, a clear separation of the bone marrow compartment and the periosteum from the soft tissue compartment of the bone defect was observed (Fig. 1c, 3 weeks, dashed line). We thus speculated that the specific ECM pattern that initially develops in critical-size segmental bone defects functions as a physical barrier that hinders endochondral bone regeneration. Consequently, we hypothesized that we could induce EO with the aid of a highly aligned biomaterial template that aligns ECM fibers along the bone axis, and thereby supports cell recruitment and directional tissue maturation across a critical-sized bone defect.

### Pore architecture controls cell migration and ECM alignment

**Fig. 2:**
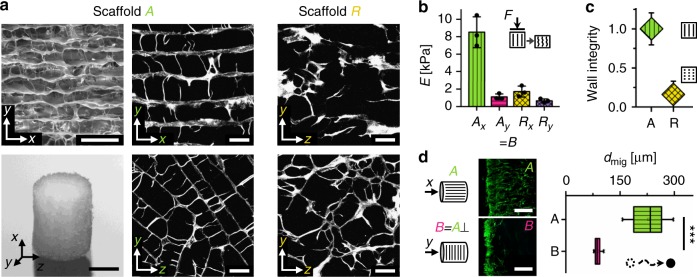
Scaffold pore architecture guides cell migration in vitro. **a** Scanning electron microscopy (SEM) image showing the scaffold pore architecture with highly aligned pores along the *x* axis (top left). Photograph of the scaffold as used in vitro (5 mm diameter, 3 mm height) (bottom left). SHG images of the scaffold pore architecture for aligned scaffolds A and random scaffold R cut along (top middle and right) and perpendicular (bottom middle and right) to the direction of directional freezing during production. **b** Mechanical stiffness of the prototypes in the two directions shown in **a** (median ± SD). Characterization was performed on *n* = 3 technical replicates. **c** Analysis of pore wall integrity indicating a pronounced reduction of pore wall integrity along the *x* direction in random scaffold R compared with scaffold A (median ± SD). Characterization was performed on *n* = 8 individual multiphoton image stacks. A high wall integrity is a characteristic feature of the channel-like pore architecture of scaffold A. **d** Quantification of the migration distance *d*_mig_ for migration of hBMSCs along (scaffold A) and perpendicular (scaffold B) to the pore orientation 3 days after cell seeding. Images on the left show representative cell distribution close to the surface of the scaffold 24 h post seeding (cross-sections stained for F-actin after cell seeding on the base of the scaffold cylinder). Significance calculated by Mann–Whitney test (two-sided). ***p < 0.001, *n* = 8 biological replicates (hBMSCs from 8 different donors), *n* = 2–3 technical replicates per donor. Boxplot in **d** shows the median, 25th and 75th percentile values (vertical bar, left and right bounds of the box), whiskers indicate the 1.5-fold IQR; open squares indicate means; crosses represent maximum/minimum values. Scale bars for **a** 200 µm in SEM image, 2 mm in photography, 100 µm in SHG images; **d** 250 µm

To guide cell migration and to control ECM patterning, we engineered a macroporous scaffold with highly aligned channel-like pores (scaffold A) from porcine collagen (Fig. 2a). A uniform pore size of 89 ± 15 µm (mean ± SD) was achieved by controlled directional freezing and subsequent freeze-drying^35^. Collagen was selected to produce a soft material with an elastic modulus in the low kPa range (*E*_Ax_ = 8.5 kPa compressive stiffness in direction of pores, see also Fig. 2b) mimicking the mechanical properties of the early ECM in endochondral bone development^36,37^. Scaffold B was cut from the identical raw material as scaffold A but with pores perpendicular to the cylinder axis. Scaffolds R (random) were produced from a collagen dispersion with identical collagen content by a modification of the freezing parameters to serve as a negative control with impaired structural integrity of the pore walls (Fig. 2a, c). Along with a changed pore architecture, scaffold R showed reduced stiffness (*E*_Rx_/*E*_Ax_ = 0.2 in *x* direction) and a reduced stiffness anisotropy (*E*_Ax_/*E*_Ay_ = 7.7, *E*_Rx_/*E*_Ry_ = 2.6) but comparable denaturation temperature as scaffold A (Fig. 2b and Supplementary Fig. 1a).

**Fig. 3:**
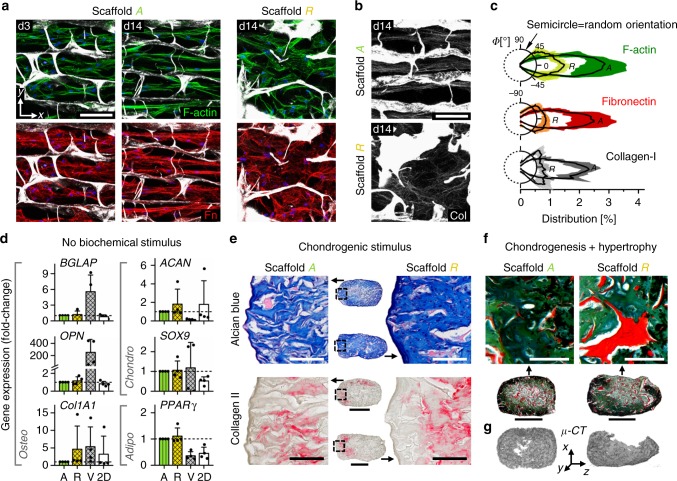
Scaffold pore architecture controls ECM structure but not cell differentiation in vitro. **a** Representative confocal images (maximal intensity projection) of hBMSC organization and matrix formation inside the scaffold pores 3 and 14 days post seeding. Top row shows F-actin of the cytoskeleton in green, lower row shows fibronectin in red. Cell nuclei are shown in blue and scaffold in gray. **b** SHG maximal intensity projections reveal formation of highly orientated collagen fibers inside the pores of scaffold A but a rather random orientation in scaffold R, 14 days post seeding. **c** Comparison of fiber orientation distribution (percent of total) for F-actin, fibronectin (Fn) and collagen (Col) in scaffold A compared with scaffold R, 14 days post seeding. Polar diagrams show mean value as solid line and standard deviation as color/gray band. *n* = 2–3 technical replicates. **d** Expression of osteogenic, chondrogenic and adipogenic genes for hBMSCs cultured on plastic (2D) and inside scaffolds A, R, and the commercial bone graft substitute Vitoss® (V). It is noteworthy that no significant differences were found between scaffolds A and R. Bar charts show fold-changes of expression compared with scaffold A (mean ± SD). *n* = 4 biological and *n* = 3 technical replicates. **e** Representative histological images of in vitro chondrogenesis of hBMSCs in scaffolds A and R over 3 weeks of culture in chondrogenic medium. Alcian blue staining (glycosaminoglycans) in top row and immunohistological staining for collagen II (red) in bottom row. No noticeable differences between the scaffold types were observed. *n* = 3 biological replicates. **f** Pronounced increase of chondrocyte volume and mineralization of the ECM indicate differentiation of scaffold-cartilage tissue into hypertrophic cartilage when cultured in hypertrophic medium for additional 2 weeks (combined Movat’s pentachrome and von Kossa staining). **g** Verification of matrix mineralization inside scaffolds by µ-CT. Scale bars for **a**, **b** 100 μm, **e** 200 µm (details) and 1 mm (overview), **f** 100 µm (details) and 1 mm (overview)

As a first step, we investigated in vitro the influence of the scaffold’s pore architecture on two essential processes for tissue regeneration: the migration of human bone marrow-derived mesenchymal stromal cells (hBMSCs) into the pores and the structural organization of the deposited ECM (fibronectin, collagen). Three days post seeding, the median migration depth was 2.7-fold higher in scaffold A (along the pores) compared with B, indicating a directional cellular access to the material (Fig. 2d). In scaffold R, almost equal migration in the different spatial directions was observed (Supplementary Fig. 1b). When hBMSCs were seeded homogeneously into scaffold A, cells and early ECM structures (fibronectin) gradually adopted a uniform alignment along the pore channels over time (Fig. 3a, c and Supplementary Fig. 1c). As of day 7, collagen fibers became visible and showed the same high structural anisotropy as fibronectin fibers (Fig. 3b,c). In scaffold R, a clearly reduced alignment of cells and ECM (fibronectin, collagen) inside the scaffold was found, but a preferential orientation in the direction of scaffold freezing during production remained (Fig. 3a–c).

The expression of bone healing-relevant genes in hBMSCs cultured inside scaffolds A and R did not show any difference demonstrating the similarity of the two materials aside of their pore architecture (Fig. 3d). Compared with two-dimensional culture on tissue culture plastic, collagen scaffolds promoted the upregulation of chondrogenic (*SOX9*) and adipogenic (*PPARγ*) genes, presumably because of the mechanically softer environment^38,39^. In contrast, Vitoss® (Stryker, USA) (scaffold V), a commercially available bone graft material used as a reference, caused strongly elevated levels of osteogenic markers osteocalcin (*BGLAP*) and osteopontin (*OPN*), while reducing chondrogenic (*ACAN*) and adipogenic (*PPARγ*) genes. Gene regulation was in agreement with the known pro-osteogenic effects of β-TCP^40^, the main component of Vitoss next to collagen.

Under pro-chondrogenic culture, hBMSCs inside scaffolds A and R showed a production of ECM rich in glycosaminoglycans (GAGs; Fig. 3e, Alcian blue staining). In addition, collagen type II was found in regions of high cell density close to the scaffold surface, indicating that cells had undergone density-controlled differentiation into chondrocytes. Genes encoding type II and X collagen were highly expressed upon induction in both scaffold types (Supplementary Fig. 1d). Subsequent culture under conditions fostering chondrocyte hypertrophy led to a noticeable increase of chondrocyte volume and successive mineralization of the surrounding ECM (Fig. 3f, g). Thus, hBMSCs were able to execute an endochondral program inside scaffolds A and R as shown previously for scaffold-free culture^20^. Cultivation of hBMSCs in scaffold A using expansion medium without pro-chondrogenic supplements before chondrogenic induction indicated that the cells preserve their differentiation potential inside the scaffold over time (Supplementary Fig. 1e).

### ECM alignment is maintained across the mineralization front

**Fig. 4:**
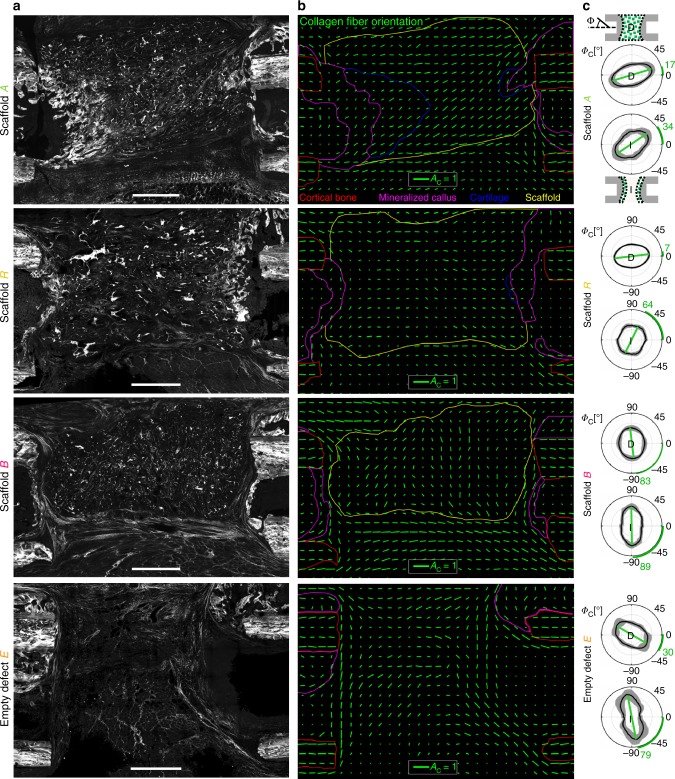
Scaffold guides collagen fibril alignment within the bone defect. **a** Representative SHG overview images of the bone defect 3 weeks post-op. **b** Analysis of local collagen fiber anisotropy in the bone defect zone for representative animals of each group. Green lines indicate anisotropy *A*_C_ (length of line) and direction of primary fiber orientation *Φ*_C_ (angle) within local ROIs of 200 µm × 200 µm size. Notice the pore orientation along the bone axis for scaffold A and perpendicular to the bone axis for scaffold B. Regions contoured by colored lines indicate cortical bone (red), mineralized callus matrix (magenta), scaffold (yellow), and cartilage (blue). **c** Distribution of collagen fiber orientation *Φ*_C_ within the scaffold (groups A, R, B) or within the empty bone defect (group E) is shown in diagrams labeled “D”. Polar plots show the distribution of orientation as mean values of all animals per group (bold line) and its SD (gray band). Bone axis is *Φ*_C_ = 0°. The fiber anisotropy can be read as the degree of polarization of the curve (long vs. short axis). Green lines and according green numbers indicate the angle of primary fiber orientation. Analysis of the local fiber orientation at the interface (diagrams labeled “I”) reveal stability of orientation along the bone axis only in scaffold A, whereas orientation in all other groups is dominated by the interface itself with fibers parallel to it and perpendicular to the bone axis (see also Fig. 1c). *n* = 5–7 animals for **c**. Scale bars for **a** 1 mm; **b** collagen fiber anisotropy *A*_*C*_ = 1

To test the potential of scaffold A to guide matrix formation in vivo, they were implanted into 5 mm critical-size segmental bone defects in rats (Supplementary Fig. 2)^41^. Scaffolds B, random scaffolds R, and empty defects E (not filled with any material) served as control groups. Via SHI, we verified a substantial impact of the pore architecture on collagen fiber anisotropy and orientation 3 weeks post-op, comparable to the in vitro findings (Figs. 4a and 3b). Fiber orientation in scaffold B was perpendicular to the bone axis, whereas collagen fiber bundles in the pores of scaffold A were spanning across the whole bone defect and had a highly consistent orientation along the bone axis. In scaffold R and empty defects E, orientation was less consistent throughout the defect. Nevertheless, a trend for collagen fiber orientation along the bone axis could still be noticed (Fig. 4b, c). This indicated an intrinsic preference for tissue alignment across the defect, abrogated only by scaffold B. However, at the mineralization front, only scaffold A was able to counteract the strong local tendency for fiber alignment parallel to the interface (perpendicular to the bone axis) as found in empty defects (Fig. 4c). Consequently, collagen fibers were observed to cross through the mineralization front in scaffold A but only sporadically in scaffold R where the fragmented walls failed to consistently control fiber alignment. This was a first indication that the scaffold’s pore architecture was able to structurally connect tissues across their various tissue maturation stages, suggesting consequences for the healing process.

### EO is controlled by scaffold pore architecture

**Fig. 5:**
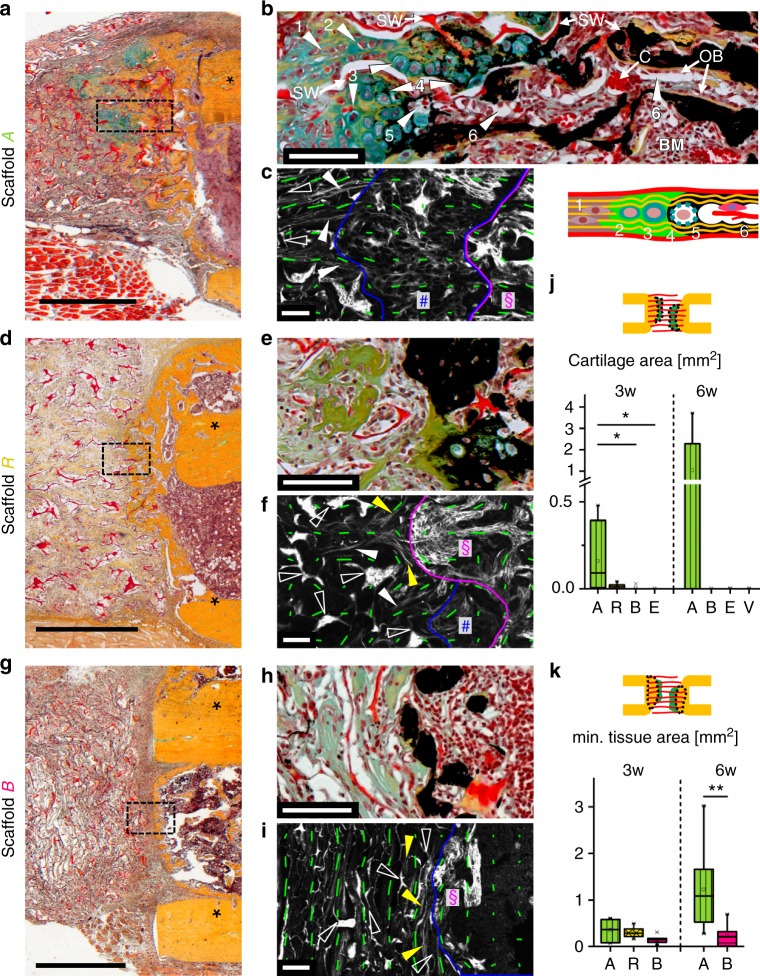
Biomaterial pore architecture is decisive for the induction of EO. **a** Movat’s pentachrome (MP) staining showing EO in scaffold A characterized by cartilage (green) at the mineralization front 3 weeks post-op. Asterisks indicate cortical bone. **b** Magnifications of the cartilage zone in scaffold A in a combined MP and von Kossa staining (mineralized matrix appears black) showing (1) pre-chondrogenic cells, (2) chondrocytes, (3) hypertrophic chondrocytes, (4) mineralization of surrounding matrix, (5) vacated chondrocyte lacunae, (6) resulting channels populated with cells and blood vessels (see also according sketch). SW indicates scaffold wall (appears red), C blood vessel capillaries, OB osteoblasts, BM bone marrow. **c** SHG image and local anisotropy (green lines) revealing consistent collagen fiber orientation during the transition from the pre-cartilage zone (left of blue line) into cartilage (between blue and magenta line, hash symbol) and mineralized matrix (right of magenta line, paragraph symbol). Full arrowheads indicate in vivo formed collagen fibrils, open arrowheads show scaffold walls. **d** Reduced ingrowth of mineralized tissue into scaffold R vs. A. Only traces of cartilage were found in individual animals (Supplementary Fig. 2e). **e** Vanishing of EO in scaffold R (3 weeks post-op) indicated by the entrapment of hypertrophic chondrocytes into mineralized matrix associated with local collagen fiber alignment parallel (**f**, yellow arrowheads). **g** Ingrowth of bone into scaffold B was found rarely. **h** Except of individual chondrocytes in one animal (Supplementary Fig. 2f), no cartilage was found at the mineralization front. **i** Collagen fibers were predominantly aligned parallel to the mineralization front. **j**, **k** Boxplots of histomorphometric data obtained from MP stainings for scaffolds A, R, B, empty defect (E), and Vitoss® (V), 3 and 6 weeks post-op with **j** total cartilage area and **k** mineralized tissue area inside the scaffold. Boxplots show the median, 25th, and 75th percentile values (horizontal bar, bottom, and top bounds of the box), whiskers indicate 1.5-fold IQR, open squares indicate means, crosses represent max./min. values. Significance via Mann–Whitney test (two-sided) with Bonferroni correction; *p < 0.05, **p < 0.01, *n* = 6–7 animals (3 weeks) and 6–8 (6 weeks) per group. Scale bars for **a**, **d**, **g** 1 mm; **b**, **c**, **e**, **f**, **h**, **i** 100 μm

Remarkably, when analyzing the tissue composition histologically, islets of chondrocytes were identified at the mineralization front within scaffold A, verifying our initial hypothesis (Fig. 5a). This result stands in strong contrast to the situation in empty defects where no signs of an involvement of chondrocytes in tissue mineralization were found. A closer look revealed that the tissue organization at the mineralization front was highly organized (see Fig. 5b): chondrocytes appeared in a columnar organization and were embedded in an aligned network of collagen fibers along the pores. Toward the mineralization front, an increased diameter indicated chondrocyte hypertrophy that was followed by gradual mineralization of the surrounding fibrocartilage matrix. Vacated chondrocyte lacunae created open channels and a high porosity within the mineralized matrix allowing vessel capillaries to advance from the bone marrow toward the mineralization front. The described processes took place along the scaffold pore walls, which finally became embedded in the mineralized matrix. Even though anisotropy was reduced in the cartilage phase, orientation of collagen fibers was maintained throughout the endochondral process (Fig. 5c). In contrast, EO in scaffold R was clearly less pronounced and only traces of cartilage were found (Fig. 5d, e). Local alignment of collagen fibers parallel to the mineralization front and perpendicular to the bone axis resulted from pore wall fragmentation and appeared to hinder EO progression (Fig. 5f). In scaffold B, consistent collagen fiber alignment parallel to the mineralization front was associated with direct deposition of mineralized tissue on the bone surface by osteoblasts, in the absence of chondrocytes (Fig. 5g–i).

Three weeks post-op, cartilage was found in five of six animals with scaffold A, in four of seven with scaffold R, in one of six with scaffold B, and in zero of six of the empty controls E. The mean area of cartilage was clearly greater in scaffold A compared with all other groups with statistical significance against scaffold B and E (Fig. 5j). Besides scaffold R that served as a negative control for structural guidance by the scaffold walls 3 weeks post-op, the commercial bone graft substitute Vitoss® (scaffold V) was used as a reference for the bone-forming ability of the scaffold 6 weeks post-op. At 6 weeks, cartilage completely filled the remaining bone gap in 3/8 animals with scaffold A, whereas no cartilage was found in the other animals of this group. No cartilage was found in any animal with scaffold B, V, or in empty controls E. The ingrowth of bone was 2.7- and 5.1-fold higher into scaffold A compared with scaffold B at 3 and 6 weeks, respectively, whereas it was 1.4-fold higher in scaffold A compared with scaffold R at 3 weeks (medians, Fig. 5k). Remarkably, in all animals EO was observed exclusively inside the scaffold. The highest total mineralized tissue area of all groups was found in specimens with ongoing EO and significantly higher values were found for scaffolds A compared with scaffold V, 6 weeks post-op (Supplementary Fig. 3a). Material degradation was comparable for scaffolds A, R, and B with a slightly stronger degradation for B compared with A at 6 weeks (Supplementary Fig. 3b). A significant reduction of scaffold area from 3 to 6 weeks post-op indicated an ongoing degradation process.

### Collagen fiber orientation is a key parameter for EO

**Fig. 6:**
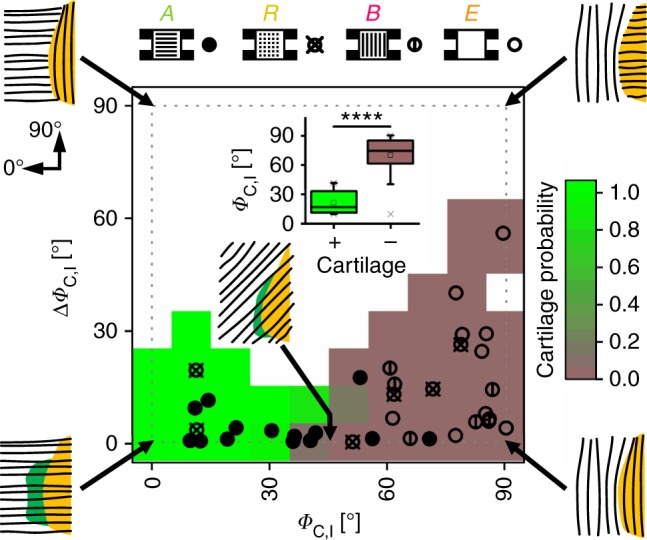
Dependency of cartilage incidence on collagen fiber orientation at the mineralization front. Data points in heatmap represent all in vivo specimens with *Φ*_C,I_ indicating the primary direction of collagen fibers in the non-mineralized matrix (0° = bone axis) and Δ*Φ*_C,I_ indicating the difference in orientation between non-mineralized and mineralized matrix. Full circles represent specimens with scaffold A, crossed circles scaffold R, circles with vertical line scaffold B, and empty circles empty controls E. Green and light brown background colors indicate high and low probability for cartilage, respectively; white background without data points. Pictograms illustrate collagen fiber orientation (black lines) at the mineralization front. Boxplot insert highlights the correlation between low values of *Φ*_C,I_ and the occurrence of cartilage. Boxplot shows the median, 25th, and 75th percentile values (horizontal bar, bottom, and top bounds of the box), whiskers indicate 1.5-fold IQR, open squares indicate means, crosses represent max./min. values. Significance via Mann–Whitney test (two-sided); ****p < 0.0001

The limited amount of cartilage in scaffold R and the low amount of bone ingrowth into scaffold B (pore orientation perpendicular to bone axis) were first indications that the scaffold’s pore architecture had a direct influence on the EO process. A closer characterization of collagen fiber orientation at the interface between non-mineralized and mineralized matrix revealed that specific structural conditions were associated with EO. The probability to find cartilage was highest if both the angle *Φ*_C,I_ of collagen fiber orientation relative to the bone axis and the deviation Δ*Φ*_C,I_ between fiber orientation in the non-mineralized compared with the mineralized matrix were small (Fig. 6). We observed that for most specimens of all groups Δ*Φ*_C,I_ was low (< 30°). This indicated a close connection between collagen fiber orientation in the non-mineralized and the mineralized matrix independent of the growth process (endochondral or intramembranous). However, a low value of *Φ*_C,I_ representing collagen fiber orientation along the bone axis was decisive for the dynamic progression of tissue mineralization via EO (Fig. 6, inserted boxplot). Such a condition was achieved most consistently in scaffold A, whereas high values of *Φ*_C,I_ were found for scaffold B and empty controls E. For scaffold R, values of *Φ*_C,I_ scattered in agreement with limited structural guidance by the fragmented pore walls leading to the sporadic occurrence of only small cartilage islands.

### Biomaterial-controlled progenitor cell and vessel recruitment

In search for the cellular origin of EO, we histologically characterized the recruitment of cells into the scaffold depending on the pore configuration. Although no unique cell surface marker for adult osteochondral progenitor cells exists, recent studies suggest CD146 as marker to discriminate between bone marrow-derived multipotent stromal cells (BMSCs) and terminal differentiated cells such as fibroblasts or trabecular bone-derived osteoblast^42^. CD146-positive (CD146+) BMSCs were shown to differentiate into cartilage and bone-like tissue in vitro^43^. In contrast to osteoblast-like cells, CD146+ BMSCs are able to form heterotopic bone ossicles and establish a hematopoietic environment upon implantation into mice^42,43^. Vessel walls in the bone marrow^44^, but also in other tissues such as the muscle^45^, were reported to act as a niche for progenitor cells.

**Fig. 7:**
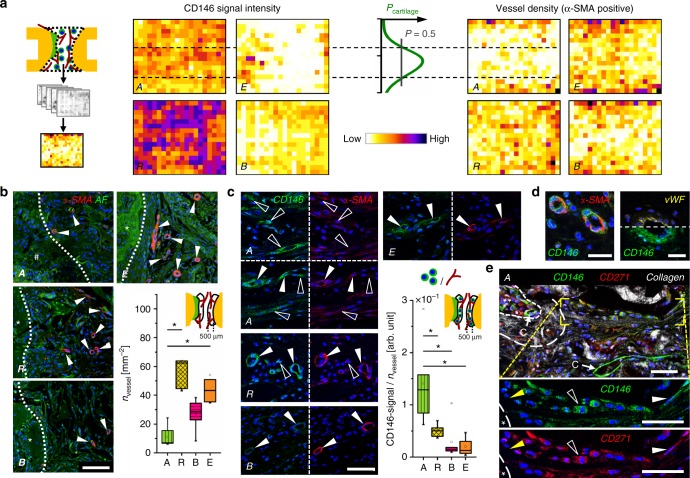
High progenitor cell number and low vessel density constitute the environment for EO. **a** Heatmaps show distribution of CD146 signal and α-SMA-positive vessel density as mean values of all animals for groups A, R, B, and E. The probability to find cartilage in scaffold A was highest in the region with low number of vessels and high number of CD146+ cells (region of *P*_cartilage_ > 50%), see also Supplementary Fig. 10. **b** Immunohistological staining for α-SMA (red), cell nuclei (blue), and autofluorescence (AF) signal (green) at the interface between cartilage (group A, hash symbol) or mineralized tissue (groups R,B,E, asterisk) and non-mineralized tissue. Boxplot shows quantification of α-SMA-positive vessels 3 weeks post-op in a ROI of 500 µm from the interface. **c** Immunohistological staining for CD146 (green) and α-SMA (red) close to the interface showing CD146 signal around vessels, but also associated with individual elongated cells for group A (open arrowheads), bright CD146 signal around vessels for group R (full arrowheads), and lower CD146 signal around vessels for group B and E. Boxplot shows the ratio between CD146 signal and vessel density 3 weeks post-op in a ROI of 500 µm from the interface. **d** CD146 and α-SMA signal were both perivascular but did not colocalize. CD146 signal was detected outside the endothelium stained by von Willebrand Factor (vWF) in consecutive sections, verifying the specificity of CD146 staining. **e** CD146+ cells were found to be involved in the process of EO. Magnifications show cells in the pre-cartilage region (full white arrowheads) and in the cartilage region (open arrowheads) expressing CD146 but also CD271. CD146 signal was also found around capillaries (C) in the pre-chondrogenic region and in the bone marrow (double asterisk). Bone marrow was enframed by mineralized tissue rich in collagen (asterisk). All boxplots show the median, 25th, and 75th percentile values (horizontal bar, bottom, and top bounds of the box), whiskers indicate 1.5-fold IQR, open squares indicate means, crosses represent max./min. values. Significances via Mann–Whitney test (two-sided) with Bonferroni correction for **b**, **c**; *p < 0.05. *n* = 5–7 animals per group for **a**, **b**, **c**. Scale bars for **b** 100 µm, **c**, **d** 50 µm, **e** 25 µm

We confirmed CD146-expressing cells around vessels in the marrow and the periosteum (Supplementary Figs. 5a and 6). Remarkably, CD146 signal in all tissues adjacent to the osteotomy (marrow, periosteum, and muscle) increased dramatically in response to the osteotomy (day 5 post-op), indicating a progenitor cell activation and subsequent recruitment to the site of injury (Supplementary Fig. 5b). In this situation, the scaffold pore architecture had a clear influence on cell recruitment into the bone defect: although low CD146 signal was found in the center of the scaffold B, an abundant number of CD146+ cells was found in scaffolds A and R (Fig. 7a, left). The high density of CD146+ cells in scaffold R can be explained by the pore wall fragmentation that permitted cell recruitment from all directions, i.e., from all adjacent tissues, whereas scaffold A was primarily accessible for cells from the bone marrow (Supplementary Fig. 7). In empty defects E, CD146 signal was restricted to a region close to the mineralization front of the bone callus. Concerning further cell types characterized, all scaffolds (A, R, B) prevented myofiber ingrowth, whereas CD68-positive cells (macrophages, giant cells) indicating a normal host response to the implanted materials were found in comparable density in the center of all scaffolds (Supplementary Fig. 8).

As oxygen supply is known to have a regulatory function in progenitor cell fate and tissue differentiation^46,47^, we next characterized the distribution of α-SMA (alpha-smooth muscle actin)-positive, mature vessels. Vessel density was low toward the axial middle line and high in the periphery for scaffolds A and B, whereas the distribution was more homogeneous in scaffold R (Fig. 7a, right). Remarkably, cartilage occurred preferentially in a central zone toward the middle axis of the scaffold that showed high CD146 signal but low vessel density (Fig. 7a, line chart and dashed lines). Motivated through this finding, we quantified vessel density and CD146 signal locally in a region of 500 µm from the cartilage or from the mineralization front (in samples without cartilage) toward the center of the defect. In this region, vessel density was lowest for scaffold A, significantly higher for scaffold R and empty controls E and intermediate for scaffold B, 3 weeks post-op (Fig. 7b). This is in agreement with the different accessibility of the materials for vessel ingrowth from surrounding tissues (muscle and periosteum) (Supplementary Fig. 7). In line with gradual scaffold degradation, vessel density equilibrated for all groups until week 6 post-op (Supplementary Fig. 3c). When the ratio between CD146 signal and vessel density was calculated, significantly higher values were found for scaffold A compared with all other groups (Fig. 7c, boxplot). The data coincided well with both the quantity of cartilage (Fig. 5j, 3 weeks) and the amount of mineralized callus grown into the scaffold (Fig. 5k, 3 weeks). In scaffolds B, and in empty controls, CD146 signal was predominantly associated with a perivascular cell location in spatial vicinity of, but not colocalizing with α-SMA signal (Fig. 7c, d). However, in scaffold A, and to a lesser extend in scaffold R, CD146+ spindle-like cells were also found distant from any vessels in the pre-cartilage region (Fig. 7c). We concluded that there are two sources for CD146+ cells that invade into the scaffold: (i) pericytes entering the scaffold from surrounding muscle and the periosteum accompanied by blood vessel ingrowth and (ii) perivascular BMSCs migrating into the scaffold from the bone marrow without pronounced vessel ingrowth. Consequently, only the physical barrier of the scaffold walls in group A, which opposes vessel ingrowth from surrounding tissues and supports migration from the bone marrow, was able to create a pro-chondrogenic environment low in vessels but rich in CD146+ cells. Moderate cell proliferation and limited cell density changes at the cartilage front supported the assumption that EO within scaffold A was primarily based on the chondrogenic differentiation of osteochondral progenitor cells and not on chondrocyte proliferation (see Supplementary Fig. 4). Finally, we found chondrocytes progressing into hypertrophy during EO to be histologically positive not only for CD146 but also for CD271, indicating that the cell source for EO may indeed be BMSCs originating from the perivascular niche^48^ (Fig. 7e).

### Structured EO leads to directional bone healing

**Fig. 8:**
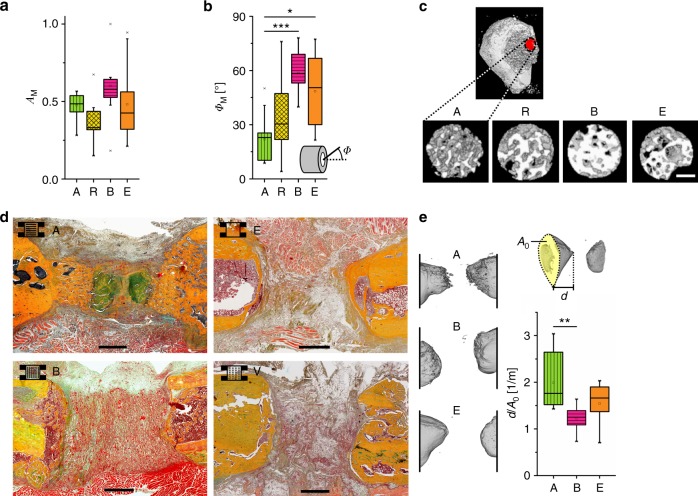
Scaffold-induced endochondral ossification forms an aligned network of mineralized matrix supporting directional bone regeneration. **a** Quantification of the structural anisotropy *A*_M_ in the mineralized matrix close to the mineralization front between non-mineralized and mineralized matrix derived from 3D reconstructed in vitro µ-CT data. Both the proximal and the distal mineralization front were analyzed resulting in two values per sample. Boxplot shows a non-significant trend for a reduced anisotropy *A*_M_ in scaffolds R compared with the other scaffold groups (A, B), 3 weeks post-op. **b** Analysis of the primary direction of anisotropy of the mineralized matrix. Boxplot shows the angle *Φ*_M_ relative to the bone axis. Significant differences were found between scaffold A and both, scaffold B and the empty control group while the data points of group R showed a strong scattering. **c** 3D µ-CT reconstruction of the mineralized matrix in the VOIs used for quantification in **a** and **b**. The red dot indicates the position of the VOI at the growth tip of the mineralized callus. **d** Movat’s pentachrome staining showing guided EO along the scaffold pore orientation for A and no ingrowth for B and V compared with the formation of a bony shell for empty controls E, 6 weeks post-op. **e** 3D µ-CT reconstruction of the resulting bone geometry for A, B, and E, 6 weeks post-op. Boxplot shows the ratio *d*/*A*_0_ representing the length of the callus in the direction of the bone axis *d* divided by the cross-sectional area *A*_0_ at the osteotomy plane as a measure of the directionality of mineralization across the bone defect. All boxplots show the median, 25th, and 75th percentile values (horizontal bar, bottom, and top bounds of the box), whiskers indicate 1.5-fold IQR, open squares indicate means, crosses represent max./min. values. Statistical significance via Mann–Whitney test (two-sided) with Bonferroni correction, *n* = 5–6 animals (**a**, **b**) and 7–8 (**e**) per group. *p < 0.05, **p < 0.01, ***p < 0.001. Scale bars for **c** 200 µm, for **d** 1 mm

In contrast to the low vessel density in the pre-cartilage zone, a larger number of α-SMA-negative capillaries reached the mineralization front from the bone marrow side in scaffold A compared with B, R, and E (Supplementary Fig. 9). In bone development, chondrocyte hypertrophy stimulates capillary ingrowth that is associated with matrix mineralization in primary ossification centers. It thus seems likely to be that α-SMA-negative capillaries, which reach the mineralization front in scaffold A, contribute to the progression of mineralization at the end region of the EO process (facing the bone marrow). According to histology and in vitro µ-CT, the morphology of the mineralized matrix was clearly affected by the scaffold’s pore architecture. The lowest value in the mean degree of anisotropy *A*_M_ of the mineralized trabecular network was found for scaffold R compared with scaffold A and B (Fig. 8a). Even though the differences did not reach statistical significance, the results are in agreement with lowest anisotropy values of collagen fiber orientation found for scaffold R (Supplementary Fig. 3d). However, highly significant differences were found in the primary orientation *Φ*_M_ of the mineralized trabecular network for scaffold A in comparison with scaffold B and empty control (Fig. 8b). Although values of *Φ*_M_ for scaffold R were strongly scattered, a very consistent trabecular alignment along the bone axis was found for scaffold A (see also µ-CT reconstruction in Fig. 8c). This suggests that the above-mentioned enhanced capillary ingrowth from the metaphysis supporting the EO mineralization process is facilitated by the aligned architecture of the mineralized trabecular network. Scaffolds B with pore orientation perpendicular to the bone axis resembled the compact mineralized matrix structure found in the empty defect.

Six weeks post-op, µ-CT showed highest volume of newly formed bone in the defect for scaffold A with bone volumes of BV = 9.3( ± 6.7) mm³ compared with 5.9( ± 2.9) mm³ for scaffold B and 5.1( ± 2.1) mm³ for empty controls (mean ± SD). A similar trend was observed for the ratio of bone volume over total volume with BV/TV = 0.24( ± 0.15) for scaffold A, 0.14( ± 0.05) for scaffold B, and 0.16( ± 0.07) for empty controls (mean ± SD). However, the variation of data within the groups was high. This was the consequence of an interference of the callus formed at the fixator pins with the callus formed within the bone defect at this time point. Morphologically, a hollow dome with a dense mineralized callus shell formed in empty controls closing the medullary cavity (Fig. 8d). In scaffold B, the medullary cavity closure happened already at the cutting plane of the osteotomy as a consequence of mineralization guided perpendicular to the bone axis along the scaffold’s surface and pore orientation (Fig. 8d). In contrast, mineralized tissue was growing in a directional manner along the bone axis in scaffold A. The finger-like morphology of the bone surface (Fig. 8e, A vs. B, E) indicated an ongoing mineralization process in those animals that were histologically characterized by a bridging of the osteotomy gap with bone and cartilage. Finally, the geometry of the mineralized callus in the defect analyzed by µ-CT confirmed a guided growth along the bone axis for scaffold A and a hindrance of bone growth into scaffold B (Fig. 8e).

## Discussion

A multitude of studies have shown that EO can be induced by combining biomaterials with progenitor cells^11,49^, by progenitor cells in combination with organic ECM components^50,51^, by pre-differentiated cells^8,52^, platelet-rich plasma^53^, growth factors, and demineralized bone matrix^54^. Progenitor cells bear an inherent potential for tissue genesis and were shown to induce ectopic EO^20^. Based on this, one might be tempted to assume that the availability of biological components (cells, factors) at the site of injury is per se not sufficient for EO to happen in large, critical-size bone defects. However, we show here that an endogenous healing cascade can be activated solely by the architecture of a biomaterial scaffold without the delivery of any additional bioactive component. EO in a critical-size bone defect was induced by controlling the structural alignment of the ECM and cell recruitment through the specific pore architecture of a macroporous biomaterial. Even though the influence of biomaterial architecture on soft and mineralized tissue organization is evident^31,32,55^, existing materials have not yet induced such a fundamental switch in the route of bone healing—from intramembranous to endochondral—as we present here.

**Fig. 9:**
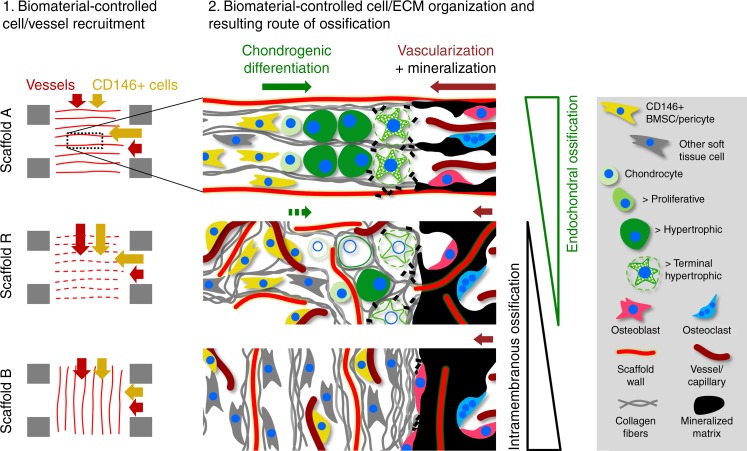
Selective cell recruitment and spatial alignment of cells and ECM induces EO inside the scaffold. Schematic illustration of the scaffold architecture-induced tissue organization and resulting ossification in the bone defect. In scaffold A, CD146+ cells are preferentially recruited from the bone marrow cavity while vessel ingrowth from the bone marrow is rare in all groups (see Supplementary Fig. 5c). Due to the pore orientation, CD146+ cell migration and vessel ingrowth from surrounding tissues is limited. Together, this creates a pro-chondrogenic condition with a high ratio of CD146+ cells per vessel. The pore architecture forces cells and ECM to align in the direction of the bone axis. At the front of EO, osteochondral progenitor cells (CD146+) differentiate into chondrocytes. Resulting from the linear alignment, vacated chondrocyte lacunae at the chondro-osseous junction form hollow channels that allow the ingrowth of capillaries (dark red arrow) stimulating terminal chondrocyte hypertrophy and migration of osteoblasts and osteoclasts remodeling the mineralized fibrocartilage matrix. Fragmented pore walls in scaffold R lead to increased invasion of vessels from surrounding tissues (muscle, periosteum), creating a less favorable environment for chondrogenic differentiation. This causes a starvation of the endochondral process. In scaffold B, perpendicular pore walls hinder migration of cells from the bone marrow space. Under these conditions, as in the empty bone defect, EO does not take place and bone formation is limited to intramembranous ossification

We propose that CD146+ BMSCs migrate from the bone marrow vascular niche along the aligned pores into scaffold A and participate in the EO process. This is supported by our observation that hBMSCs cultured inside scaffold A in vitro were capable to initially produce the characteristic, highly aligned ECM found in vivo, and subsequently execute an endochondral program under the appropriate biochemical stimuli. In vivo, the growth of vessels into the scaffold was strongly limited for scaffold A, indicating a limited oxygen supply that favors chondrogenesis of BMSCs over osteogenesis^25,56^. However, it is also known that the bone marrow niche is low in oxygen^57^ and restriction of vascularization might rather preserve the stemness of BMSCs inside scaffold A^58^. In line with this, cartilage was found only as a thin intermediate phase of EO preceding mineralization, indicating that the chondrogenic differentiation of BMSCs is tightly interlinked with the spatiotemporal progression of the EO process itself. We suggest that for initial EO to converge into a spatially well-controlled process, the gradual diffusion of signaling molecules towards BMSCs in the pre-cartilage region is required to induce and orchestrate their proliferation and chondrogenic differentiation^59^. This is supported by theoretical and experimental work that showed facilitated diffusion along aligned fiber networks compared with perpendicular or random structures^60,61^. Regarding the late events in the EO process, terminal chondrocyte hypertrophy and matrix mineralization are coupled to vascularization. Capillaries, however, can reach the chondro-osseous junction only if linearly arranged hypertrophic chondrocytes produce channel-like structures in the trabecular mineralized matrix as found for scaffold A (Fig. 5b). Together, according to our explanatory model summarized in Fig. 9, scaffold-induced ECM structuring allows EO to happen via improved communication at two important EO junctions: the transition into the cartilage phase and the transition from the hypertrophic into the mineralized matrix phase. The strong correlation between collagen fiber orientation and the occurrence of EO (Fig. 6) clearly demonstrates that scaffold-controlled ECM alignment is involved in the regulation of the EO process, whereas BMSC recruitment and vessel restriction provide the necessary environment for EO to take place. Even though we have evidence that scaffold-induced EO is also observed under increased scaffold stiffness (Supplementary Fig. 11), the robustness of the process against the variation of other material parameters such as pore size and degradation remains to be proven. The similarity of the here-reported scaffold-induced ossification with the highly aligned and spatially controlled EO in the bone’s growth plate^62^ motivate a future in-depth comparison of the maturation cascades and involved regulating factors.

In summary, we demonstrate that within the channel-like pores of a scaffold that was engineered to provide a guiding structure for ECM alignment and progenitor cell recruitment, a switch from intramembranous ossification closing the bone marrow cavities toward a directional EO across the bone defect could be achieved. Developmental engineering in vivo has recently been suggested as a method to support bone fracture repair along the endochondral route via a mimicry of the early fibro-cartilaginous ECM^6^. Our results demonstrate that tissue structure is one of the key parameters to be taken into account in such an approach. A more rigorous incorporation in biomaterial design strategies is suggested.

## Methods

### Cell isolation and culture

hBMSCs were obtained from donors undergoing total hip joint replacements. The study was approved by the ethics committee of the Charité – Universitätsmedizin Berlin and all donors gave informed written consent. hBMSCs from eight donors were used. hBMSCs were isolated by density gradient separation and subsequent adhesion to tissue culture polystyrene. Cells were cultured in medium consisting of Dulbecco’s modified Eagle’s medium (DMEM, Sigma, 1000 mg l^−1^ glucose) supplemented with 10% fetal bovine serum (FBS, Biochrom AG), 1% penicillin/streptomycin (P/S, Biochrom AG), and 1% l-glutamine (glutaMAX, Invitrogen). Medium was exchanged twice per week and the cells were trypsinized (PAA Laboratories GmbH) when a confluency of 80% was reached. Experiments were performed at passage 4–5 (differentiation) and 5–6 (migration).

Human fibroblasts (data provided in Supplementary Fig. 1b) were isolated from skin obtained from orthopedic surgeries. The study was approved by the ethics committee of the Charité – Universitätsmedizin Berlin and all donors gave informed written consent.

### Scaffold fabrication

Highly porous collagen scaffolds with aligned pores of homogeneous pore size were produced by controlled directional freezing and freeze-drying of a 1.5% (wt/wt) collagen dispersion^35,63–65^. A constant temperature gradient was initially realized between two parallel, temperature-controlled metal plates that enclose the collagen dispersion (thickness: 10 mm, area of mold: 100 × 100 mm). Then, the temperature of both plates was lowered at the same constant rate (Power-Down technique). After ice nucleation at the colder plate, a stable ice front develops (constitutional supercooling) and ice crystals grow parallel in finger-like morphology through the collagen dispersion with a constant ice front velocity defined by the applied cooling rate. Collagen is pushed between the ice crystals creating aligned, channel-like, pores after freeze-drying (scaffolds A and B). The final pore size and orientation is defined by the finger-like ice crystal morphology which is determined by the combination of applied temperature gradient (1 K mm^−1^) and cooling rate (0.01 K s^−1^). Random pore architecture (scaffold R) was realized by freezing in absence of controlled ice crystal growth and morphology. Scaffolds were crosslinked using 1-ethyl-3-(3-dimethyl aminopropyl)carbodiimide hydrochloride, freeze-dried, and sterilized via Ethylene Oxide treatment (HA2 Medizintechnik GmbH, Germany). The mean pore size was evaluated as the average distance between pore walls on microscopic images of multiple sections of the bulk material. Stiffness was characterized on three to four individual specimens via a monoaxial compression test using a BOSE ElectroForce TestBench system (TA Instruments ElectroForce Systems Group). The denaturation temperature, indicating the degree of crosslinking and the stability against degradation, was analyzed via a Differential Scanning Calorimeter Q100 (TA Instruments, USA).

### Second harmonic generation imaging of collagen fibers

Collagen fibrils exhibit endogenous second harmonic generation (SHG) signals arising from their well-known non-center-symmetric molecular structure^66,67^. SHI was performed on a Leica SP5 II microscope using a × 25 water immersion objective with a numerical aperture of 0.95. The SHG signal was generated by a Spectra Physics Ti:Sapphire laser (Mai Tai HP) with 100 fs pulse width at 80 MHz and wavelength of 910 nm. SHG signal from collagen was detected at 450–460 nm (half excitation wavelength).

### Analysis of scaffold pore wall integrity

SHG images of empty scaffolds wetted with phosphate-buffered saline (PBS) were binarized and scaffold wall integrity was analyzed based on the length of the individual walls in *x* direction (according to coordinates indicated in Fig. 2a). ImageJ plug-in AnalyzeSkeleton was used to quantify maximum length of the wall segments in each image (Longest Shortest Path). Walls with a length > 310 µm (half image width) were regarded as intact walls. Data were normalized to the number of intact walls found in scaffold A.

### 3D cell migration into collagen scaffolds

To create a cell layer, 2 × 10^5^ cells (cell suspension of 2000 cells µl^−1^ in expansion medium) were seeded in the center of a custom-made silicone rings with an inner diameter of 7 mm within a 12-well plate. After 1 h of incubation, the silicone ring was removed and the dense layer of attached MSCs was washed twice with PBS to eliminate non-adherent cells. Cylindrical scaffolds (type A, B and R, 5 mm diameter, 3 mm height) were pre-wetted in expansion medium and placed on top of the cell layer to allow cell migration into the scaffold and 800 µl of medium were carefully added. Pre-wetting was important to avoid that loosely-attached cells were soaked into the dry scaffold. After 24 h, scaffolds were detached from the well, washed twice with PBS and transferred to new wells. It was ensured that the cell layer was on the bottom side during the whole experiment. Finally, 1.3 ml of serum-free medium consisting of DMEM supplemented with 1% P/S, 1% non-essential amino acids (NEA, Biochrom AG), and 1% Nutridoma-SP (Sigma) was added. Scaffolds were fixed 72 h post transfer in 4% paraformaldehyde (PFA). Cryosections of 25 µm thickness were prepared from the region around the midplain of the scaffold and stained for cell nuclei (4′,6-diamidino-2-phenylindole, DAPI). Overview images were recorded via a fluorescence microscope (Zeiss Axioskop 40) and positions of nuclei relative to the scaffold surface were analyzed via digital image analysis using ImageJ software. The median of the migration distance perpendicular to the surface was calculated for each scaffold from at least two images. For hBMSCs, quantification was performed on eight biological replicates for the comparison between scaffold A and B (orientation perpendicular to A) (Fig. 2b) and additionally on two biological replicates for the supplementary comparison between A, B, R, and RT (orientation perpendicular to R) (Supplementary Fig. 1b). Supplementary data for hDFs were assessed on one biological replicate.

### 3D ECM formation in collagen scaffolds

Scaffolds of cylindrical shape (*D* = 4 mm, *h* = 3 mm) were seeded homogeneously by dipping them into a cell suspension of 10,000 cells µl^−1^ for MSCs and 7500 cells µl^−1^ for fibroblasts (supplementary data, lower cell density for fibroblasts to compensate the higher proliferation rate). Scaffolds were placed into individual wells of a 12-well plate without additional medium. After 1 h of incubation scaffolds were washed in PBS to remove non-adherent cells. Scaffolds were transferred to new wells and 1.4 ml of medium were added. Medium was DMEM (1000 mg l^−1^ glucose for BMSCs, 4500 mg l^−1^ for fibroblasts) with 10% FBS, 1% Glutamax (BMSCs only), 1% P/S, 1% NEA (fibroblasts only), and ascorbic acid (0.23 mM for BMSCs, 1.36 mM for fibroblasts) (Sigma). Medium was exchanged twice a week. Cylindrical scaffolds were fixed in 4% PFA and cut along the cylinder axis to be imaged for fibrillar collagen (SHI, for details see imaging of in vivo samples). Subsequently, the same samples were stained for cellular actin filaments (Alexa fluor 488 Phalloidin, #A12379, Thermo Fisher), fibronectin (anti-fibronectin antibody, #ab23750, Abcam plc), and cell nuclei (DAPI, #D3571, Thermo Fisher) for imaging via confocal microscopy. Linear LUTs (lookup tables) covering the full data range were used for representation.

### Analysis of in vitro cell and ECM fiber anisotropy

The degree of cell (F-actin cytoskeleton) and ECM (fibronectin) anisotropy of in vitro samples was analyzed from maximum projections of confocal image stacks (60 µm stack depth, 4 µm z-plane spacing). Image stacks were recorded with a Leica SP5 II confocal laser scanning microscope (Leica Mikrosysteme Vertrieb GmbH, Wetzlar, Germany) equipped with a × 25 water immersion objective. Collagen fibers were visualized via SHG imaging (for details see in vivo histology). Distribution of fiber orientation was analyzed via the ImageJ plug-in OrientationJ (by Daniel Sage) on two to three technical replicates (scaffolds) and three confocal image stacks per scaffold. Time dependency of cell and ECM anisotropy was evaluated from two biological and two to three technical replicates for hBMSCs (Supplementary Fig. 1c).

### Gene expression of BMSCs cultured inside collagen scaffolds

BMSCs isolated from four different human donors were used. Influence of the scaffold material on gene expression was evaluated by seeding 2500 cells µl^−1^ into cylindrical scaffolds (*D* = 5 mm, *h* = 3 mm) as described above. Cell-seeded scaffolds were cultured for 7 days in expansion medium (DMEM with 1000 mg l^−1^ glucose, 10% FBS, 1% Glutamax, 1% P/S). Total RNA isolation and purification from cell-seeded scaffolds was performed using PureLink^®^ RNA Mini Kit (Thermo Scientific) in combination with the On-column PureLink® DNase Kit (Thermo Scientific). Subsequently, mRNA was transcribed into cDNA (iScript^TM^ cDNA Synthesis Kit [Bio-Rad]) and quantitative PCR was performed based on SYBR green. Mean normalized expression ratios, using *HPRT* as the reference gene, were calculated using the efficiency corrected ∆∆Ct method and fold change expressions were determined in comparison to the control. Four biological and three technical replicates were included in the analysis. Primer sequences for the genes of interest are listed below.

Osteocalcin (*BGLAP)*: forward 5′-TGAGAGCCCTCACACTCCTC-3′, reverse 5′-CGCCTGGGTCTCTTCACTAC-3′

Osteopontin (*OPN)*: forward 5′-CACTACCATGAGAATTGCAGTGA-3′, reverse 5′-CTGCTTTTCCTCAGAACTTCCA-3′

Collagen typ I, alpha1 (*Col1A1)*: forward 5′-AGCCGGAGATAGAGGACCAC-3′, reverse 5′-GGCCAAGTCCAACTCCTTTT-3′

Aggrecan (*ACAN)*: forward 5′-GGGTTTTCGTGACTCTGAGG-3′, reverse 5′-ATGGGGTCGATGAAATAGCA-3′

*SOX9*: forward 5′-GGAGACTTCTGAACGAGAGCG-3′, reverse 5′-CCGTTCTTCACCGACTTCCTC-3′

*PPARγ*: forward 5′-TGCAGTGGGGATGTCTCATA-3′, reverse 5′-CAGCGGGAAGGACTTTATGT-3′

*HPRT*: forward 5′-TATGGACAGGACTGAACGTC-3′, reverse 5′-TGATGTAATCCAGCAGGTCA-3′

For data provided in Supplementary Fig. 1d:

Collagen typ II, alpha1 (*Col2A1)*: forward 5′-CTGGAAAAGATGGTCCCAAA-3′, reverse 5′-CAGGGAATCCTCTCTCACCA-3′

Collagen typ X, alpha1 (*Col10A1)*: forward 5′-CCCAACACCAAGACACAGTTC-3′, reverse 5′-AGGACTTCCGTAGCCTGGTT-3′

### Chondrogenic differentiation of BMSCs in vitro

Cell seeding density was increased to 10,000 cells µl^−1^ and scaffold volume was reduced (*D* = 2 mm, *h* = 3 mm) to support chondrogenic differentiation of BMSCs. Scaffold were pre-cultured in BMSC expansion medium. Following 1 day or 14 days of pre-culture (collagen fiber formation), scaffolds were incubated for 21 days in serum-free chondrogenic differentiation medium consisting of DMEM (4500 mg l^−1^ glucose) supplemented with 1% P/S, 1% GlutaMAX™ (Gibco), 100 nM dexamethasone (Sigma)^68^, 10 ng ml^−1^ transforming growth factor (TGF)-β1 (Peprotech)^68^, 50 µg ml^−1^ (0.23 mM) l-Ascorbic acid 2-phosphate (Sigma), 40 µg ml^−1^ (0.35 mM) l-Proline (Sigma), and 0.1 mg ml^−1^ (0.91 mM) sodium pyruvate (AppliChem).

To induce chondrocyte hypertrophy, scaffolds were further cultivated for 14 days in serum-free hypertrophic medium. Medium was chondrogenic differentiation medium with removal of TGF-β1 and sodium pyruvate, reduction of dexamethasone to 1 nM and addition of 10 nM T3(3,3′,5-Triiodo-l-Thyronine) (Sigma), 50 pg ml^−1^ IL1-β (Peprotech)^69^, and 10 mM β-glycerophosphate (Sigma)^69^.

Specimens were fixated (4% PFA) and mineralization of specimens cultured under hypertrophic conditions was analyzed via µ-CT (Skyscan 1172 F, Bruker) with 4.98 μm isotropic resolution at 80 kV source voltage and 124 μA source current. Subsequently, the cylindrical specimens were cryo-cut to the half-diameter and 10 µm cryosections were prepared. Sections were stained for Alcian blue (GAGs), collagen type II (staining details see in vivo histology, no pretreatment) and Movat’s pentachrome combined with von Kossa staining for visualization of chondrocyte hypertrophy and mineralization. Collagen fibers were visualized via SHG imaging on the flat cutting surface of the specimens after cryo-sectioning to the mid-plane of the cylindrical scaffolds.

### Animals

The study was approved by the local authorities (LaGeSo Berlin, G 0415/12 and G 419/12). Sixty-four female Sprague–Dawley rats with an average weight of 290 g at day of surgery were randomly divided into eight groups (four groups A, B, R, E, to be killed after 3 weeks and four groups A, B, V, E, 6 weeks). The rats were purchased from Charles River Deutschland GmbH, Germany, had free access to food and water and were kept in the animal facility for at least 7 days before surgery. All animal experiments were carried out according to the policies and procedures established by the Animal Welfare Act, the NIH Guide for Care and Use of Laboratory Animals, and the National Animal Welfare Guidelines. The study was approved by the local authorities. Animal number per group was chosen based on a statistical power-analysis, mandatory for the approval of the animal study. In the study four animals died as a consequence of anesthesia for µ-CT imaging; five animals were suffering from bone lysis at the fixator pins and were killed ahead of schedule; further two animals were excluded from analysis because of pin lysis. One additional animal was excluded because the scaffold was pushed out of the defect due to forces from surrounding tissues (histological evaluation).

### Animal surgery

The rats were administered an intraperitoneal injection of ketamine hydrochloride (60 mg kg^−1^, ketamine 50 mg, Actavis, Island) and medetomidine (0.3 mg kg^−1^, Domitor, Pfizer, Karlsruhe), as well as the antibiotic clindamycin (45 mg/kg, Ratiopharm, Ulm) subcutaneously. Using an anterio-lateral approach the femur was exposed by bluntly separating the gluteus superficialis and biceps femoralis muscles. The external fixator (RatExFix, RISystems, Davos) with bending stiffness of 254 N mm^−1^ (ExFixHigh)^70^ was mounted by four titanium (Ti-6Al7Nb) threaded pins manually screwed into the femur after predrilling each pin hole. A 5 mm femoral defect into the mid shaft of the femur was created by performing a double transverse osteotomy. Scaffolds (type A, B, R, V) of cylindrical shape (4 mm diameter, 5.5 mm height) were wetted in saline solution and inserted into the bone defect (slight press-fit due to 10% oversize). Scaffolds were labeled with numbers to blind the veterinarian during surgery. For empty controls, the gap remained unfilled. Fascia and skin were sutured and the analgesic tramadol hydrochloride (20 mg kg^−1^, Grünenthal, Aachen) was administered subcutaneously and for a period of three days diluted in the animals’ drinking water. To recover from anesthesia the animals received an intraperitoneal injection of the antidote (1.5 mg kg^−1^, Antisedan, Pfizer, Karlsruhe). Immediately after surgery, the rats were allowed to resume normal activity and given unrestricted access to food and water again.

Radiographic images were taken at week 2, 3 (3 week animals), and at week 2, 4, 6 (6 weeks animals) following the same anesthesia protocol as described above. In vitro µ-CT was performed after sacrificing the animals at week 3 or 6. All animals were given numbers to blind the investigators during data analysis of the different groups.

### Histological staining and evaluation of in vivo samples

Animals were killed at the indicated time points, femora were dissected and fixated in 4% PFA for 48 h at 4 °C. Samples were washed and placed into PBS before in vitro µ-CT was performed. Subsequently, femora were quick-frozen in liquid nitrogen and cut along the long axis in the plane of the pins of the external fixator using a custom-made sample holder and a low-speed saw (IsoMet, Buehler GmbH, Germany). One half was placed into 15% ethylenediaminetetracetic acid (EDTA, Herbeta Arzneimittel, Germany) for decalcification and subsequent embedding in paraffin, the other half was dehydrated in an ascending ethanol row for embedding in polymethylmethacrylate (PMMA).

SHI for visualization of collagen fibers was performed on the remaining PMMA blocks of embedded samples after preparation of histological sections. Overview images of the bone defect were recorded using a Leica SP5 II microscope equipped with a × 25 water immersion objective with a numerical aperture of 0.95 (tile scan). SHG signal was generated by a Spectra Physics Ti:Sapphire laser (Mai Tai HP) with 100 fs pulse width at 80 MHz and wavelength of 910 nm. SHG signal from collagen was detected at 450–460 nm. For quantification of collagen fiber orientation, single z-planes at a depth of 4 µm or 8 µm from the surface of the PMMA block were selected.

Autofluorescence signal (AF) was recorded via two-photon microscopy at 800 nm laser wavelength excitation and detection at 380–680 nm. AF imaging was performed on a Leica SP5 II microscope using a × 25 water immersion objective with a numerical aperture of 0.95.

All imaging data obtained from confocal or two-photon microscopy was represented using linear LUTs covering the full data range.

Movat’s pentachrome staining was performed on 4 µm paraffin sections for discrimination of tissue types (collagen in bright yellow, cartilage in blue-green, GAGs bright blue, osteoid dark red, elastic fibers red, cytoplasm light red, and nuclei brown black). A combination of von Kossa staining (visualization of calcium deposits in black) and Movat’s pentachrome staining was performed on 7 µm PMMA sections (heavy duty microtome) for information on tissue types and tissue mineralization.

Immunohistological stainings for Ki-67 and CD68, as well as combined stainings CD146/CD271, CD146/von Willebrand Factor (vWF), and CD146/α-SMA were performed on 4 µm paraffin sections. For Ki-67, heat-induced epitope retrieval (waterbath, 8 min, 90 °C) was performed in TRIS-EDTA-buffer at pH 9. Primary anti-Ki-67 antibody (rabbit monoclonal, #ab16667, Abcam) was used at 1:25 dilution. For CD68, primary anti-CD68 antibody (mouse monoclonal, #BM4000, Acris) was used at 1:2000 dilution. For combined CD146/CD271 and CD146/α-SMA staining, heat-induced antigen retrieval was preformed using citrate buffer at pH 6 (3 min, pressure cooker). Primary anti-CD146 antibody (rabbit monoclonal, #ab75769 clone EPR3208, Abcam) was used at 1:300 dilution, primary anti-CD271 antibody (mouse monoclonal, #MAB365 clone 192-IgG, Millipore) at 1:30 dilution, and primary anti-α-SMA antibody (mouse monoclonal, #ACTA2 clone 1A4, Dako) was used at 1:400 dilution. For vWF, pretreatment was 0.1% pepsin in 0.01 N HCl (15 min, room temperature (RT)). Primary anti-vWF antibody (rabbit polyclonal, #CP039B, Biocare) was used at 1:200 dilution. All primary antibodies were diluted in Antibody-diluent (DAKO) and incubated overnight at 4 °C. For detection via bright-field microscopy (avidin/biotin complex), biotinylated secondary antibodies (2%, 30 min, RT), Alkaline-Phosphatase Standard-Kit (50 min, RT), and Alkaline-Phosphatase-Substrate Kit (incubation time controlled under microscope) were used (all from VECTOR Laboratories, Inc.). Methylgreen (VECTOR) was used for counterstaining of nuclei in Ki-67 stainings (25 min, RT), and hematoxylin (Mayer’s) in CD68 and α-SR1 stainings. Sections were imaged via bright-field microscopy using a Zeiss Axioskop 40 (Ki-67, CD68) or via confocal microcopy using a Leica SP5 II confocal microscope (CD146, CD271, vWF, α-SMA). Histomorphometric quantification of scaffold, cartilage, and mineralized matrix area were performed via digital image analysis using ImageJ/Fiji software and custom-made data analysis macros (available from corresponding author upon request). For detection via confocal microscopy, secondary antibodies used were CY3 (anti-rabbit, #711-165-152, Dianova) at 1:200, Alexa Fluor® Plus 647 (anti-mouse, #A32728, Thermo Fischer) at 1:200, and Alexa 488 (anti-mouse, #A-11001, Thermo Fischer) at 1:400 dilution. Cell nuclei were visualized by DAPI (#D3571, Thermo Fisher) at 1:1500 dilution (15 min, RT).

Immunohistological staining for α-SMA (quantifications shown in Fig. 7a,b) and α-SR1 were performed on 5 µm PMMA sections. Primary anti-α-SMA antibody (mouse monoclonal, #ACTA2 clone 1A4, Dako) was used at 1:400 dilution (overnight, 4 °C). For α-SR1, heat-induced antigen retrieval was preformed using citrate buffer at pH 6 (30 min, waterbath at 90 °C). Primary antibody was anti-α-SR1 (mouse monoclonal, #ab28052, Abcam) at 1:50 dilution. For detection via bright-field microscopy, the avidin/biotin complex was used as described above (Supplementary Fig. 8b). For immunofluorescence (α-SMA stainings shown in Fig. 7b, Supplementary Figs. 5c and 9), a secondary antibody coupled to Alexa Fluor® 488 (anti-mouse, #A-11001, Thermo Fischer) was used at 1:400 (1 h, RT). DAPI was used for counterstaining of nuclei at 1:1500 dilution (15 min, RT). Signal from α-SMA and nuclei were recorded via confocal microscopy. In addition, AF signal was recorded via two-photon microscopy at 800 nm laser wavelength excitation and detection at 380–680 nm. α-SMA-negative capillaries in Supplementary Fig. 9 were detected by the presence of erythrocytes (anuclear + bright AF signal from hemoglobin) in vascular structures.

All antibodies were tested on reference tissues reported to serve as positive controls. Results were evaluated by an experienced histologist. Negative control stainings were performed without using the primary antibody.

For heatmap representation of vessel density, CD146, CD68, and α-SR1 signal intensity distribution (Fig. 7a and Supplementary Fig. 8), regions of interest (ROIs) were manually selected to cover the bone defect region for empty defects E and to cover the scaffold area within the defect region for groups A,R,B. Regions of mineralized tissue, cartilage, and muscle prolapsed into the bone defect were excluded from the analysis. All ROIs were stretched to a uniform rectangular shape using a customized macro in ImageJ using the plug-in “Landmark Correspondences” by Stephan Saalfeld. Vessels were marked manually for each animal in each group. Intensities of CD146, CD68, α-SR1 signal were summed for all animals within the respective group creating 32 bit images. Mean vessel densities and mean signal intensities (CD146, CD68, α-SR1) within sub-ROIs (20 × 16 squared sub-ROIs) were calculated, normalized to the number of animals per group and represented in a mosaic-heatmap-format using ImageJ. Preprocessing of the images in ImageJ was cell nuclei and background-subtraction (thresholding) for CD146 and background-subtraction (thresholding) for CD68 and α-SR1.

For the analysis of local blood vessel density and local CD146 signal intensity at the callus front (boxplots in Fig. 7b, c), ROIs of 500 µm from the interface between mineralized and non-mineralized tissue toward the direction of the non-mineralized tissue were defined. The ROIs were restricted to the bone defect region for group E and the scaffold region within the bone defect region for groups A,R,B excluding muscle tissue. Cartilage (avascular) was excluded from the analysis. In regions of cartilage, the interface between cartilage and non-mineralized tissue (preceeding cartilage) rather than the interface between mineralized and non-mineralized tissue was used as boarder for the ROI (see also sketch in Fig. 7c). Blood vessels (showing a lumen, positive for α-SMA) within the ROIs were counted manually from bright-field images using ImageJ and mean blood vessel density *n*_vessel_ was calculated from the number of vessels divided by the area of the ROI. Mean CD146 signal intensity in the ROIs was analyzed from confocal microscopy images using ImageJ (following image processing described above).

### In vivo collagen fiber orientation and anisotropy

Primary orientation and local anisotropy of collagen fibrils in in vivo samples was analyzed from SHG images using the freely accessible ImageJ plug-in FibrilTool^71^. FibrilTool provides information about the primary orientation and the degree of anisotropy of a fiber network. FibrilTool was implemented into a macro performing orientation analysis within multiple sub-ROIs of defined size within a grid-like pattern covering the osteotomy region (used for Fig. 1c: sub-ROI size 45 × 45 µm, Fig. 4b and 5c, f, i: sub-ROI size 194 × 194 µm). A green line in each sub-ROI indicates the degree of anisotropy (*A*_C_ = length of line, 0 < *A*_C_ < 1) and direction of primary orientation (*Φ*_C_ = angle of line relative to horizontal orientation).

To quantify the distribution of collagen fiber orientation inside the bone defect (Fig. 4c, polar diagrams labeled “D”), ROIs were manually selected to cover the scaffold area for groups A,R,B and to cover the bone defect region for empty defects E. Regions of mineralized tissue, cartilage and muscle were excluded from the analysis. Fiber orientation at the interface (Fig. 4c, polar diagrams labeled “I”) was quantified in manually selected ROIs of 320 µm width (in the direction of the bone axis) across the full height of scaffold/bone defect. Distribution of fiber orientation was analyzed via the plug-in OrientationJ, normalized to 1 (divided by the sum of values for 0 to 360°) and plotted as polar diagrams. The direction of primary fiber orientation, indicated by green lines and according numbers in Fig. 4c, was analyzed as the maximum of a Gaussian distribution fitted to the distribution of fiber orientation depending on the angle *Φ* in Cartesian coordinates.

For the correlation between collagen fiber orientation and occurrence of cartilage (Fig. 6), fiber orientation on both sides of the interface between the mineralized and non-mineralized matrix was analyzed using FibrilTool. ROIs of 320 µm width (in the direction of the bone axis) and 1300 µm height (perpendicular to bone axis) were manually selected at the tip of the mineralized callus on the proximal and the distal side of the segmental bone defect. The incidence of cartilage depending on fiber orientation was evaluated from AF images and Movat’s pentachrome staining on histological sections.

### In vitro µ-CT

For analysis of mineralized matrix anisotropy and mineralized callus shape, in vitro µ-CT was performed at 3 and 6 weeks post-op (vivaCT 40, Scanco Medical, Bassersdorf, Switzerland), respectively. Femora were scanned using 10.5 μm isotropic resolution, with 55 kV source voltage, 145 µA source current, and 150 ms integration time. The volume of interest (VOI) was defined to include the 5 mm defect region and 0.5 mm in the proximal and distal directions from the cutting plane of the bone defect. A global threshold of 60% (callus shape and mineralized matrix anisotropy) relative to the mineral density of the intact tibia, equivalent to 351 mg HA ccm^−1^ was used to distinguish mineralized tissue from poorly mineralized and unmineralized tissue.

### Analysis of mineralized ECM orientation and anisotropy

Cylinders of 705 µm diameter and 378 µm height (VOIs) were cropped out from in vitro µ-CT image stacks at the tip of the proximal and distal mineralized callus via ImageJ. Position of the VOIs was defined as follows: cylinder axis = bone axis, cylinder base = first plane and position where a circle of 705 µm diameter is filled by mineralized callus starting from the middle of the bone defect, cylinder end = startplane + 378 µm. Two VOIs were extracted from each in vivo specimen (proximal and distal callus tip). Thresholding was applied to gray scale at 60% cortical bone gray value. BoneJ plug-in (http://bonej.org) was used for the analysis of anisotropy *A*_M_ on binarized image stacks and primary direction of anisotropy *Φ*_M_ was calculated from the eigenvector with smallest according eigenvalue.

### Analysis of mineralized callus shape

Based on reconstructed µ-CT data, height of the mineralized callus *d* and the cross-sectional callus area *A*_0_ at half callus height were analyzed to compare geometry of the newly formed mineralized bony callus between the scaffold groups and the empty control group. Callus height was defined as the distance between the osteotomy plane and the tip of the mineralized callus (callus tip = first plane where the cross-sectional area was ≥ 0.20 mm² when moving from the callus tip into the callus). The ratio *d*/*A*_0_ gives information about the shape of the callus. At comparable total callus volumes, directional bone healing across the defect (cylindrical shape) is represented by higher values of *d*/*A*_0_ compared with a non-directional pattern (half sphere-like shape).

### Analysis of cell density at the cartilage front

Imaging of collagen fibers and cell nuclei were performed directly on PMMA blocks to avoid structural artefacts and associated alterations of cell density due to sectioning. DRAQ5 (1:500) was pipetted on the block in the osteotomy region and incubated for 1 h at RT. Blocks were washed with PBS (3 × 15 min). Confocal and multiphoton image stacks were recorded for cell nuclei (DRAQ5) and collagen fibers (SHI), respectively (see Supplementary Fig. 4b). Position of cell nuclei were manually marked on maximum projections of image stacks. Distribution of nuclei along the bone axis (*x* axis) was quantified in a histogram with a class width of 150 µm. Cell density was calculated from the number of cells per interval divided by the area (image height x class width). Data were analyzed from four specimens (scaffold A) and is provided as Supplementary Fig. 4c.

### Statistics

All statistical analysis was performed in Origin Pro 2015G (OriginLab Corporation). In vivo experiments were replicated at least twice (animal surgery performed at different days). No significant difference between the results was found. In vitro experiments were repeated at least twice with identical results. The exact number of repeats for in vitro experiments are given in the figure captions. Details of the statistical analysis are provided in Supplementary Table 1.

## Electronic supplementary material

Supplementary Information

Peer Review File

## Data Availability

The authors declare that the data supporting the findings of this study are available within the paper and its Supplementary Information files.
