# Supplementary material for: A biomaterial with a channel-like pore architecture induces endochondral healing of bone defects
Source: Nat Commun. 2018 Oct 25;9:4430. doi: 10.1038/s41467-018-06504-7 (PMC6202397; doi:10.1038/s41467-018-06504-7)
Supplement: Supplementary file 1 — Supplementary Information [file 41467_2018_6504_MOESM1_ESM.pdf]

Nature Communications

Supplementary information for

A biomaterial with a channel-like pore architecture induces endochondral healing of bone defects

Petersen A. et al.

Correspondence and requests for materials should be addressed to ansgar.petersen@charite.de

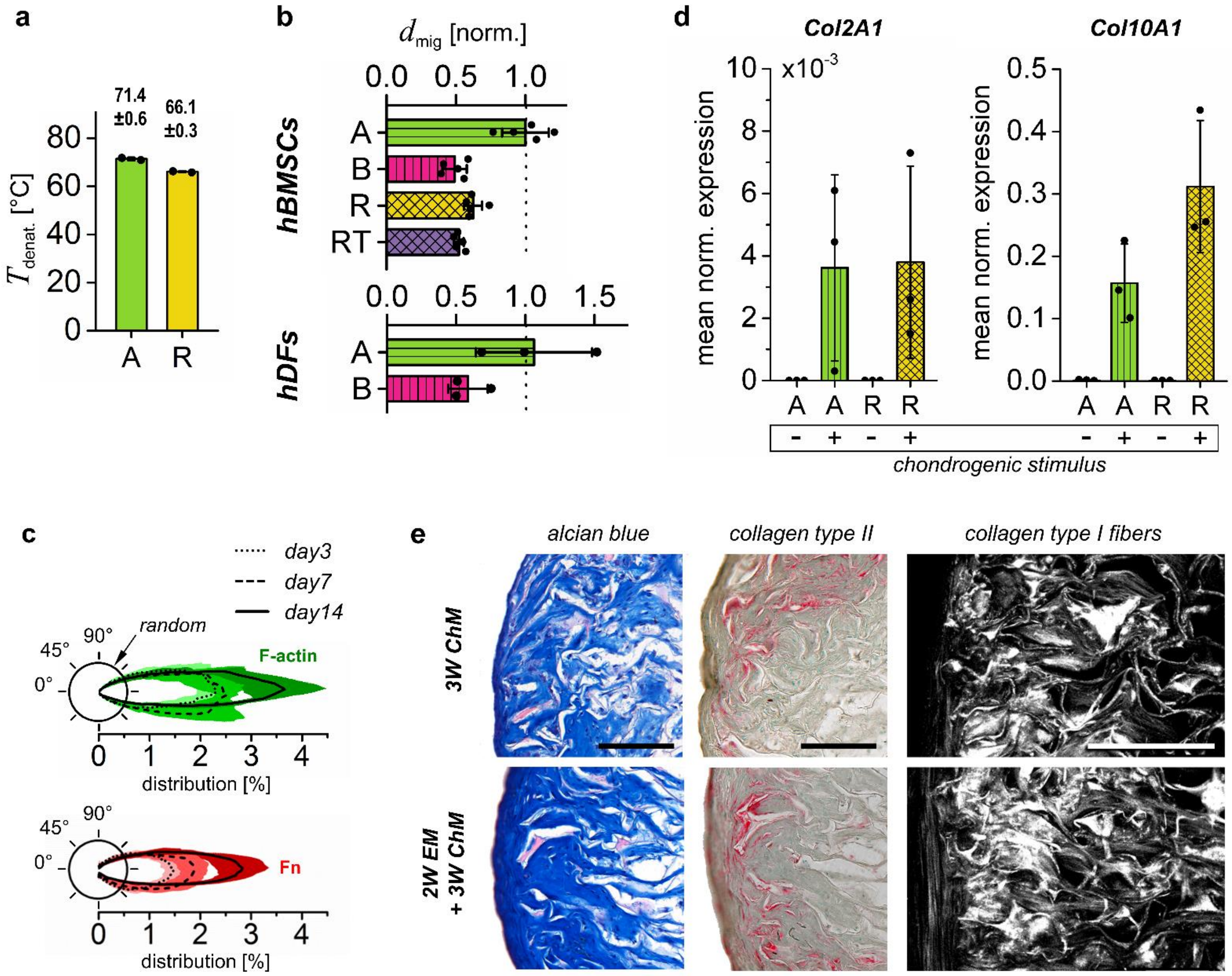

Supplementary Figure 1 Complementing information on scaffold material and scaffold-guided cell migration, differentiation and organization in scaffold pores. **a** Denaturation temperature measured via differential scanning calorimetry (mean±SD). Values for Scaffold A and B are identical since they were cut from the same raw material. **b** Comparison of cell migration into scaffolds A and R along the direction of primary pore orientation and perpendicular to it (B, RT), normalized to scaffold A. Boxplots show the median of the migration distance d_{mig} for hBMSCs and hDFs, 3 days after cell seeding. The preferential migration along pores of scaffold A is lost for fragmented pore walls of scaffold R. Fibroblasts and hBMSCs show similar capacity for migration into the scaffold. n=2 biological and 2-3 technical replicates for hBMSCs and n=3 technical replicates for hDFs. **c** Scaffold pore architecture guides matrix formation. Time-dependency of F-actin and fibronectin (Fn) fiber orientation (percent of total) for hBMSCs in scaffold A. Polar diagrams show data for day 3, 7 and 14 with mean value as solid line and standard deviation as color band. n=2 biological and n=2 technical replicates per timepoint. **d** Gene expression of hBMSCs in scaffolds A and R showing the upregulation of type II and X collagen after incubation for 7 days in chondrogenic differentiation medium compared to expansion medium (mean±SD). n=3 biological and 2 technical replicates. **e** *In vitro* chondrogenesis by hBMSCs in scaffolds A and R. Direct chondrogenesis over 3 weeks in chondrogenic medium (ChM) containing TGFβ (top row) was compared to scaffolds that were pre-cultured for two weeks in expansion medium (EM) followed by culture in chondrogenic medium for additional 3 weeks. Both conditions generated cartilage-like tissue close to the scaffold surface (histology for alcian blue and collagen II) while collagen fibers were more pronounced in the latter situation (SHI). Scale bars in **e** 200 µm.

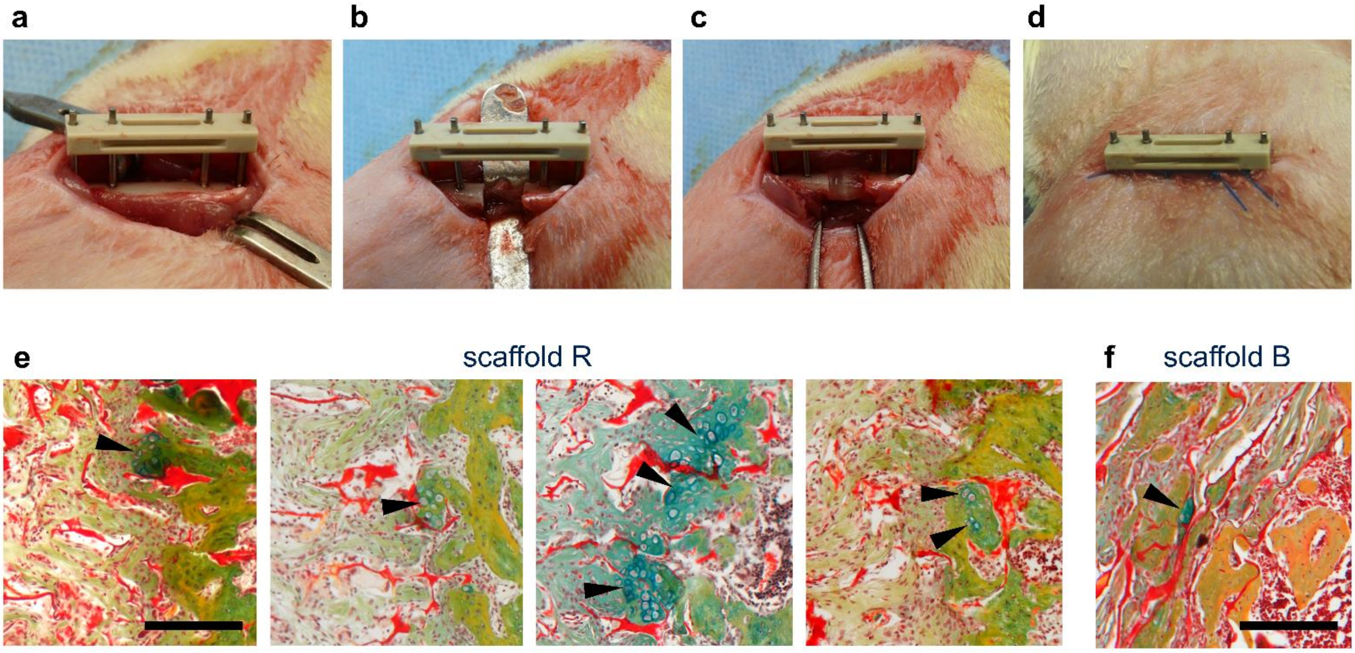

Supplementary Figure 2 Animal model with scaffold implantation during the surgical procedure and complementing histological images. **a** Mounted external fixator, **b** critical size defect (5 mm) created in the rat femur, **c** implantation of the scaffold fully filling the gap and **d** closure of the wound. **e** Movat Pentachrome stainings showing small islands of chondrocytes (arrowheads) in 4/7 animals with scaffold R. **f** Individual chondrocytes (indicted by arrowhead) found in 1/6 animals with scaffold B. Scale bars 200 µm.

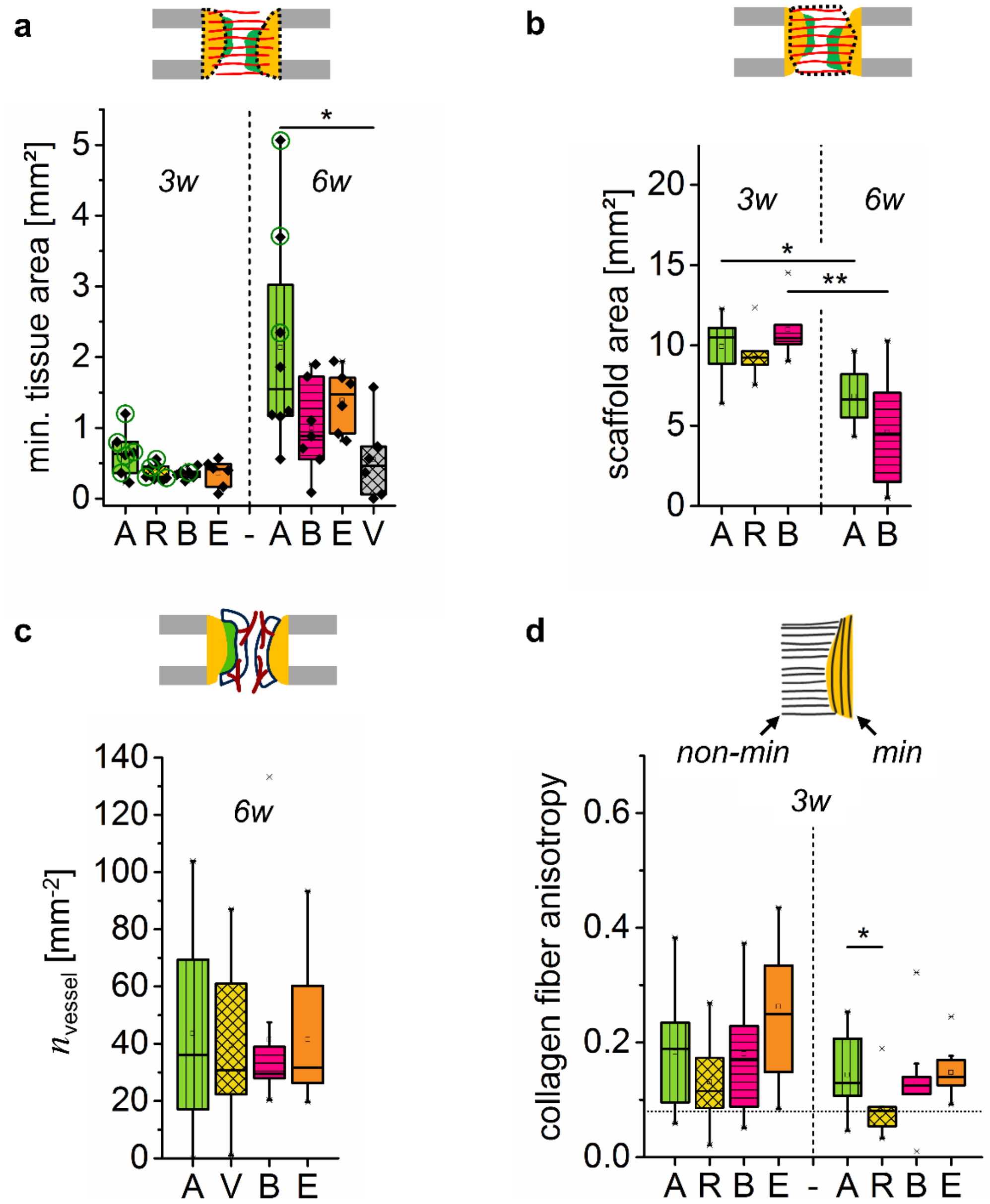

Supplementary Figure 3 Complementary histological evaluations. **a** Total mineralized tissue area in the fracture gap based on histomorphometric analysis (Movat's pentachrome staining) 3 and 6 weeks post-op represented by boxplots (complementing Fig. 5k). Diamond symbols indicate data points of individual animals, green circles highlight samples where cartilage was found indicating bone formation via EO. For scaffolds A and R those samples represented the animals with the highest values of newly formed bone. non-min = non-mineralized; min = mineralized tissue matrix. n= 6-7 animals (3 weeks) and 6-8 (6 weeks) per group. **b** Boxplots of histomorphometric data obtained from Movat's pentachrome stainings showing total scaffold area as a measure of scaffold degradation 3 weeks post-op for scaffolds A, R and B and 6 weeks post-op for scaffolds A, B. n= 6-7 animals (3 weeks) and 6-8 (6 weeks) per group. **c** Complementary data for histological analysis of vascularization. Boxplots representing vessel density (α-SMA-positive vessels) for scaffolds A, R, B and empty defects E 6 weeks post-op supplementing the data shown in Fig. 7b. No difference was found between the scaffold groups at this time point. n= 6-8 animals per group. **d** Boxplots show collagen fiber anisotropy at the mineralization front 3 weeks post-op, supplementing the data of fiber orientation shown in Fig. 6. n=5-7 animals per group. All boxplots show the median, 25th and 75th percentile values (horizontal bar, bottom and top bounds of the box), whiskers indicate 1.5-fold IQR, open squares indicate means, crosses represent max./min. values. Significance via Mann-Whitney Test (two-sided) with Bonferroni correction; *$p<0.05$, **$p<0.01$.

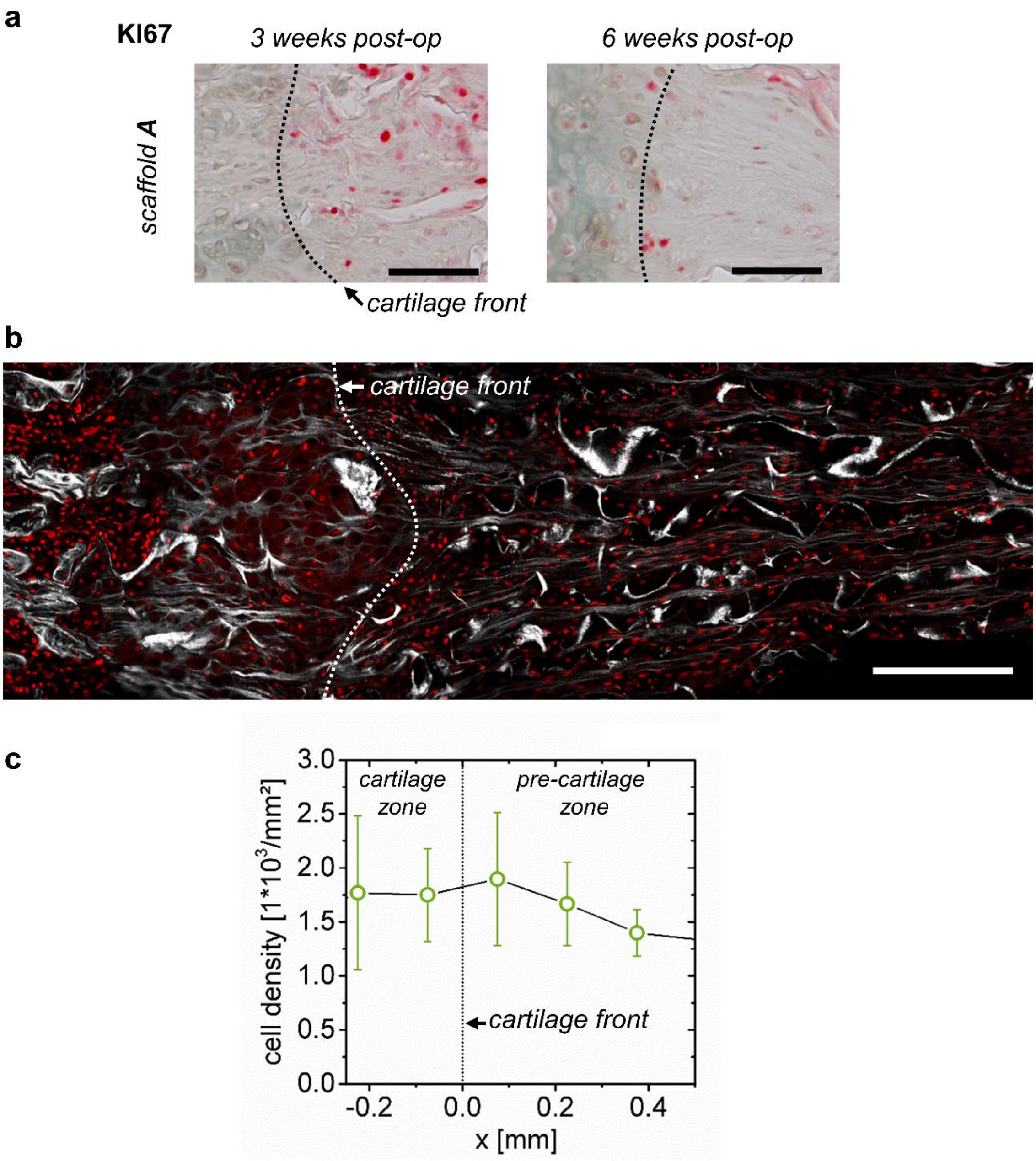

Supplementary Figure 4 Complementary histological evaluation of cell proliferation and density at the cartilage front. **a** KI67 proliferation marker staining 3 and 6 weeks post-op showing only individual positively stained nuclei in the pre-cartilage zone. Non-stained nuclei indicate resting cells in the G(0)-Phase. **b** Overlaid SHG-signal (fibrillar collagen, white) and nuclei (DRAQ5, red) showing cell density at the cartilage front. **c** Histomorphometric quantification of cell density on PMMA-embedded specimens as shown in **b** (DRAQ5-stained PMMA blocks) revealed a slight increase in the pre-cartilage phase in agreement with KI67 staining. n=4 biological replicates. Data points indicate mean values, error bars indicate standard deviation. Scale bars in **a** 75 µm, **b** 200 µm.

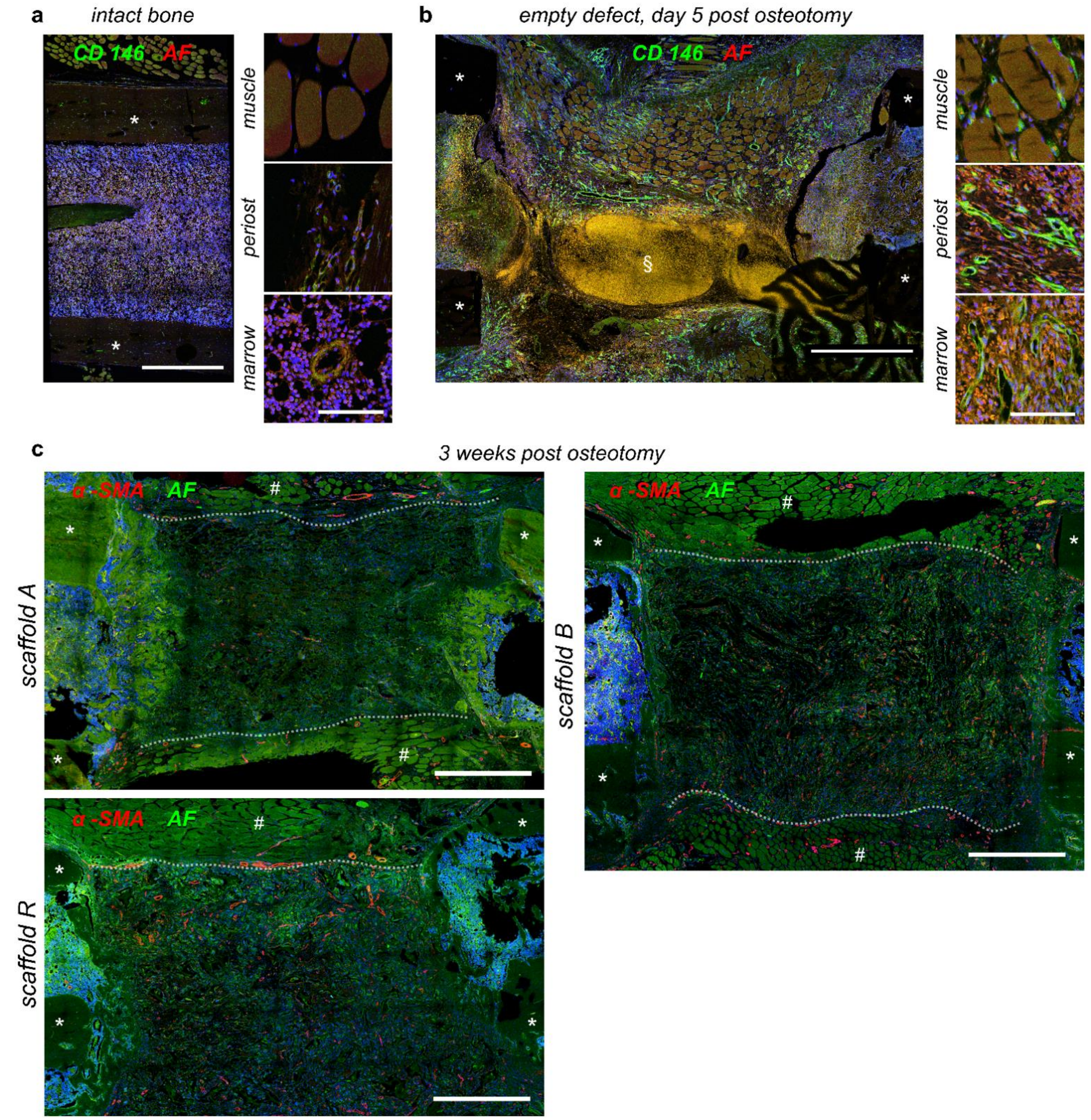

Supplementary Figure 5 Histological overview images showing CD146 cell recruitment and bone defect vascularization 3 weeks post-op. **a** In intact contralateral bones low CD146 signal was found around vessels within the bone marrow, and within muscle and periosteum surrounding the bone. **b** 5 days post osteotomy, CD146 signal in all adjacent tissues was strongly increased indicating CD146+ cell recruitment to the site of injury. **c** Immunohistological staining for scaffolds A, B and R, highlighting α-smooth muscle actin (α-SMA) positive vessels in red, autofluorescence signal (AF) in green and cell nuclei stained by DAPI in blue. Erythrocytes in the bone marrow show bright green but no blue signal (no nucleus). Unmineralized tissue in the scaffold and muscle tissue can be distinguished by tissue morphology based on autofluorescence signal. Depending on pore orientation and pore architecture, α-SMA-positive vessels are predominantly guided along the surface of the scaffold (scaffold A) or penetrate deep into the scaffold (scaffold B and R). Vessel ingrowth from the bone marrow is strongly limited. Paragraph symbol for hematoma, asterisks for cortical bone, hash symbols for muscle tissue. Scale bars in **a** (left), **b** (left), **c** 1 mm; in magnifications **a** (right) and **b** (right) 100 µm.

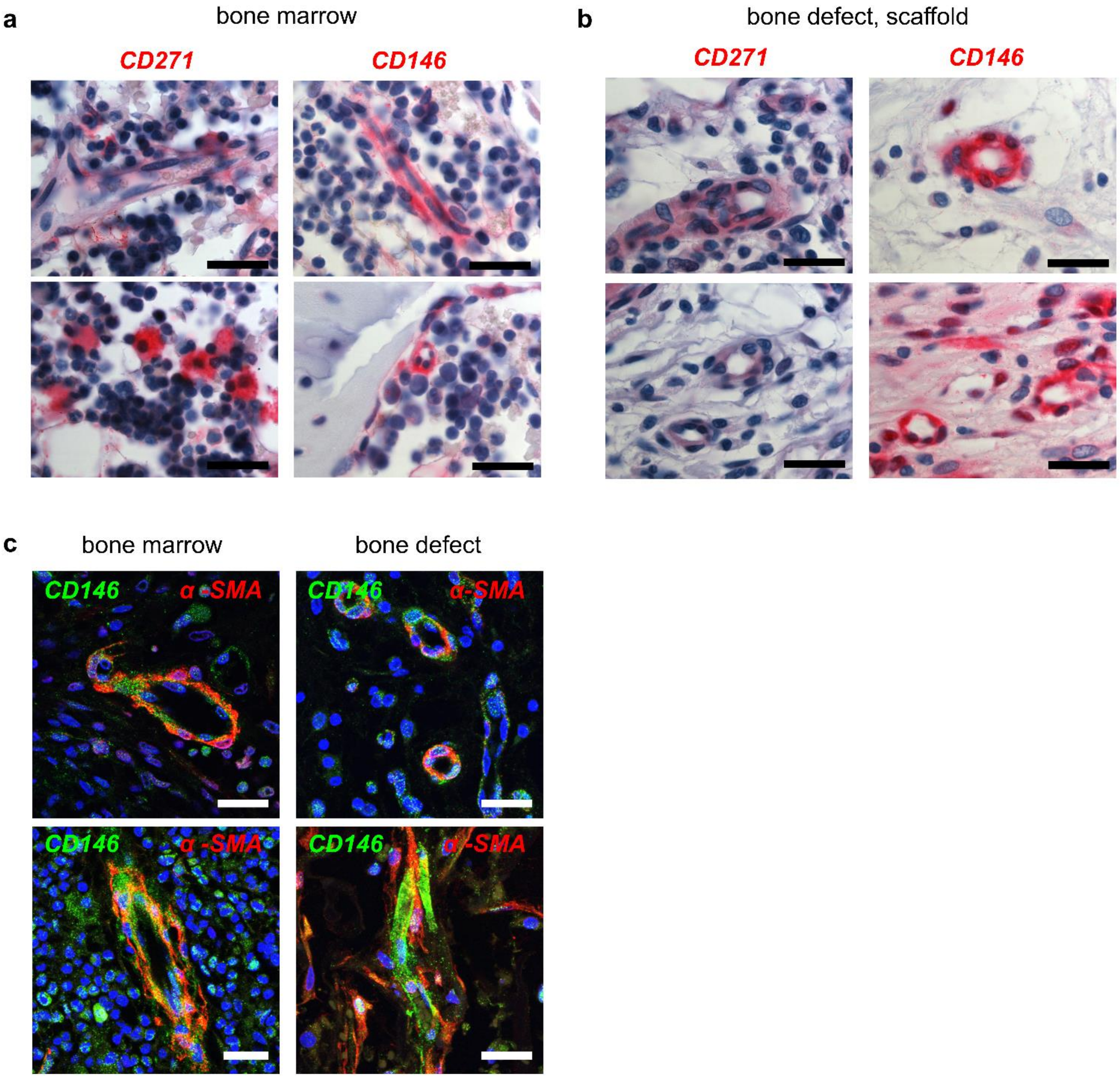

Supplementary Figure 6 Complementary histological stainings showing the specificity of CD146 and CD271 signal in bright field and fluorescent immunohistology. **a** In the bone marrow CD146+ cells were found surrrounding vessels and CD271+ cells were found as polygonal cells throughout the marrow and as elongated cells around vessels. **b** Within the scaffold, perivascular cells showed strong CD146 signal and weak CD271 signal. **c** CD146 perivascular signal found within the bone marrow and in the scaffold is spatially close to but not identical with a-SMA signal. All scale bars 25 µm.

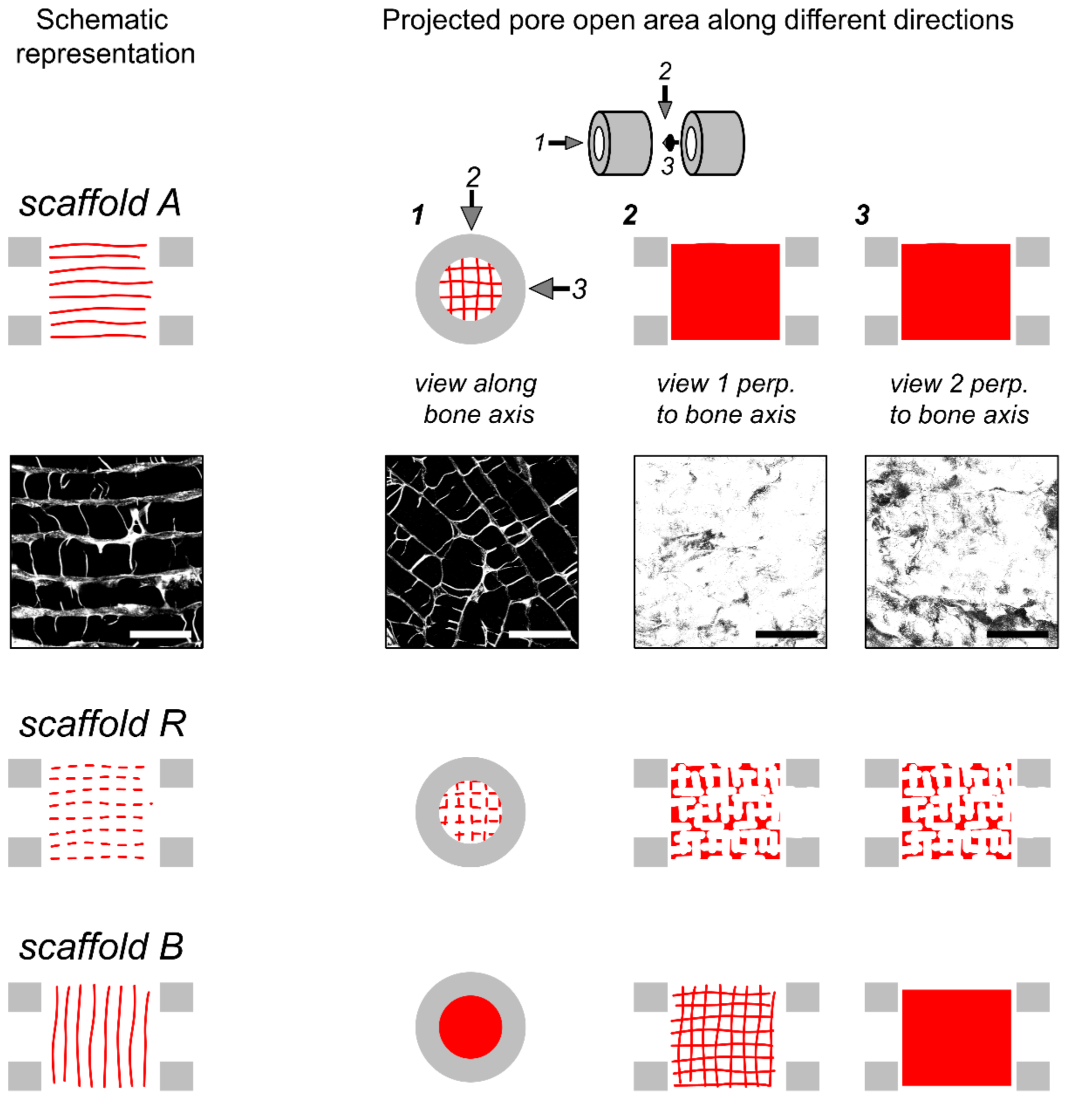

Supplementary Figure 7 Sketch indicating the projected open pore area for scaffold A, B and R along the bone axis and two perpendicular directions. Scaffold R has the overall highest open pore area as scaffold walls are fragmented. This explains higher number of blood vessels inside scaffold R compared to scaffold B. Even though scaffold B supports cell invasion from surrounding tissues along the direction of the pores, it blocks invasion in the direction perpendicular to it (and perpendicular to the bone axis). Maximum intensity projection images of SHI showing the scaffold's pore architecture as a slice through the open pores (left) and the according open pore area as deep image stacks from two perpendicular directions (right) according to the sketch. Scale bar 200 µm.

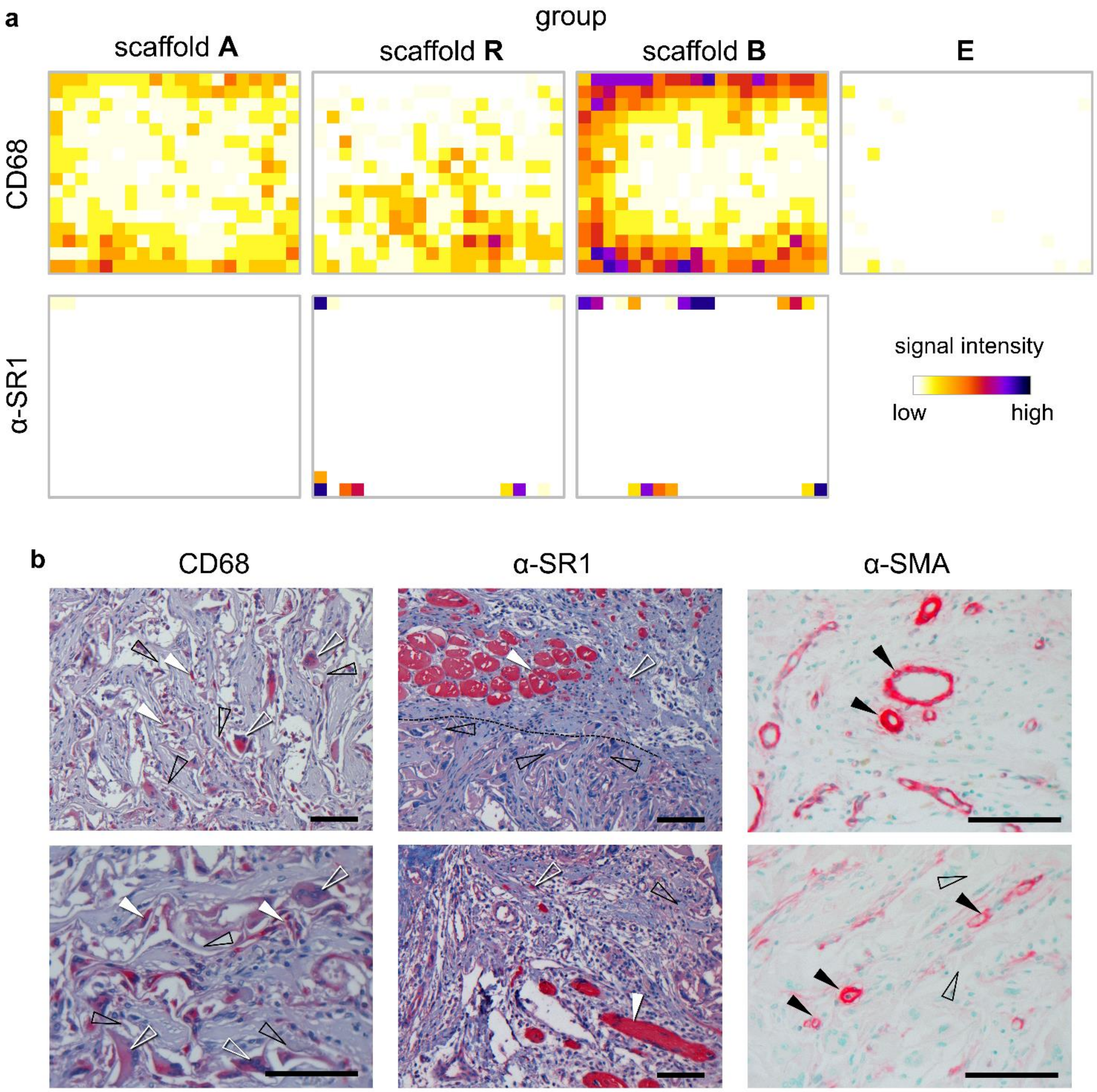

Supplementary Figure 8 Analysis of cell distribution within the osteotomy region. **a** Distribution of CD68-positive cells (macrophages, giant cells) within scaffolds and empty defects and α-SR1-positive myofibers within the scaffold A, R, B supplementing the data shown in Figure 7a. **b** Exemplary histological images for CD68, α-SMA and α-SR1 used for cell/vessel identification. Open black arrowheads for CD68, α-SMA and α-SR1 indicate scaffold walls. For CD68 full white arrowheads exemplarily indicate CD68-positive cells with single nuclei (e.g. macrophages), open white arrowheads indicate multinuclear giant cells within the scaffold. For α-SR1, open white arrowheads exemplarily indicate newly developing myofibers and full white arrowhead indicate mature myofibers in skeletal muscle at the border of the scaffold. For α-SMA full arrowheads indicate vessels of different sizes within the scaffold (bottom) and surrounding tissue (top). Scale bars in **b** 100 µm.

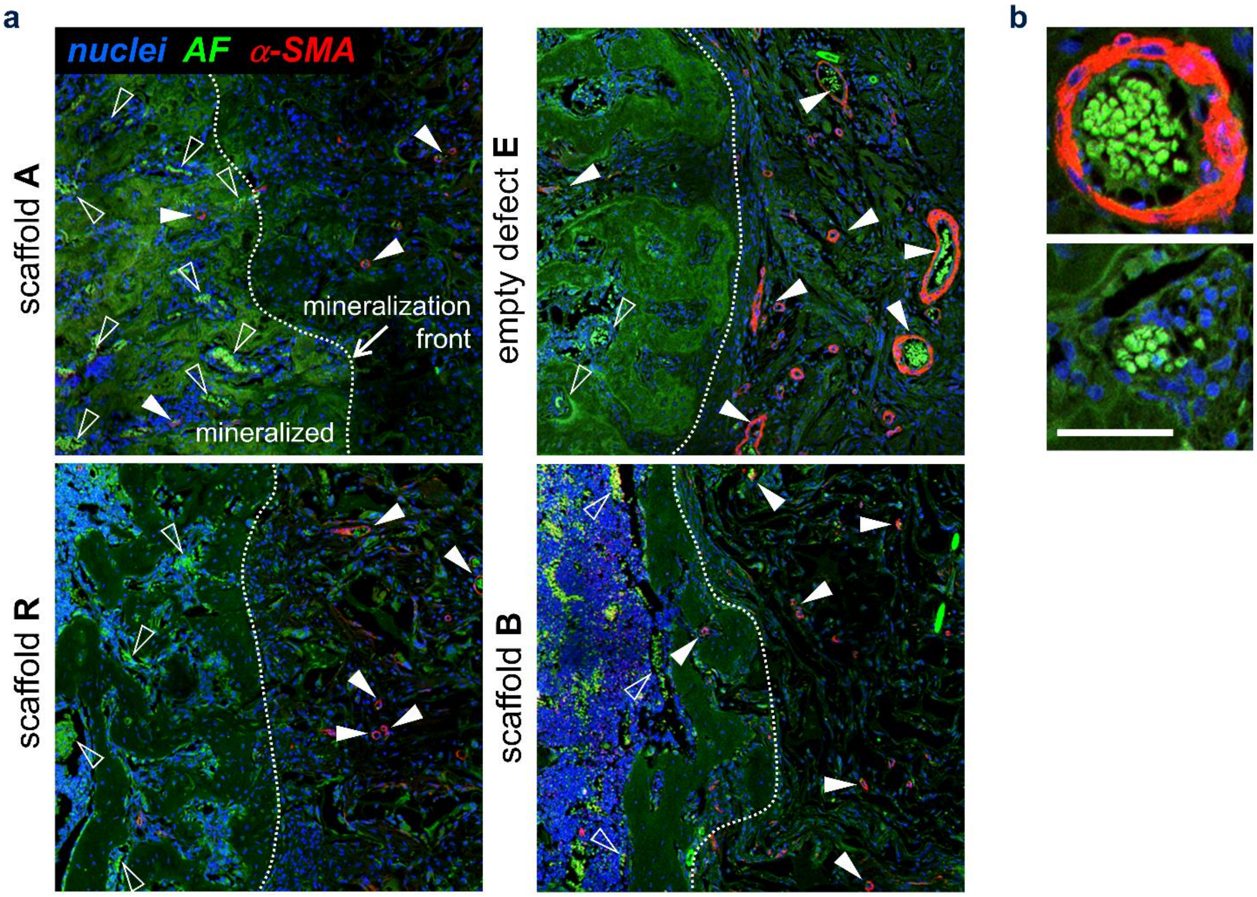

Supplementary Figure 9 Visualization of blood vessel distribution at the mineralization front for scaffolds A, R, B and the empty defect (E). **a** Immunohistological staining for α-smooth muscle actin (α-SMA) in red, cell nuclei in blue and autofluorescence signal (AF) in green. Full arrowheads indicate α-SMA vessels, open arrowheads indicate α-SMA-negative capillaries. **b** Anuclear erythrocytes in α-SMA-negative capillaries and α-SMA-positive vessels show bright green but no blue signal (magnifications). Scale bars 200 µm.

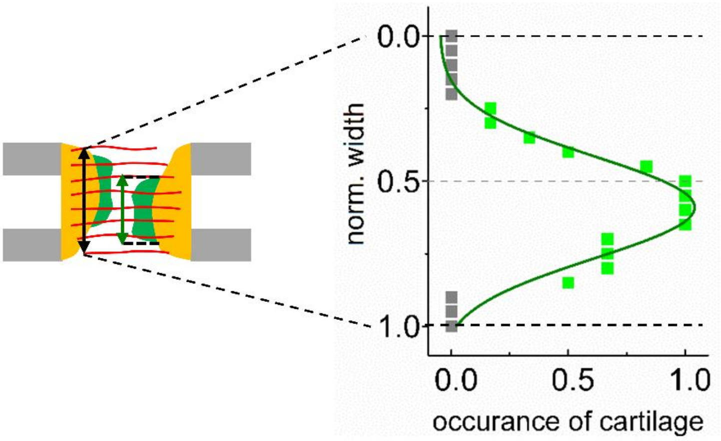

Supplementary Figure 10 Spatial analysis of the occurrence of cartilage within scaffold A. Frequency plot showing a preferential *in vivo* occurrence of cartilage in a zone close to the middle-axis of the scaffold (0.5 normalized width). Scaffold boundaries at 0 and 1.0 norm. width. Data was obtained via histomorphometry from animals with cartilage in scaffold group A, 3 weeks post-op (n=5 animals). The lateral extension of cartilage was measured at the height of the mineralization front (see sketch) and normalized to the scaffold width of each specimen. Curve fitted via bi-Gaussian function to guide the eye.

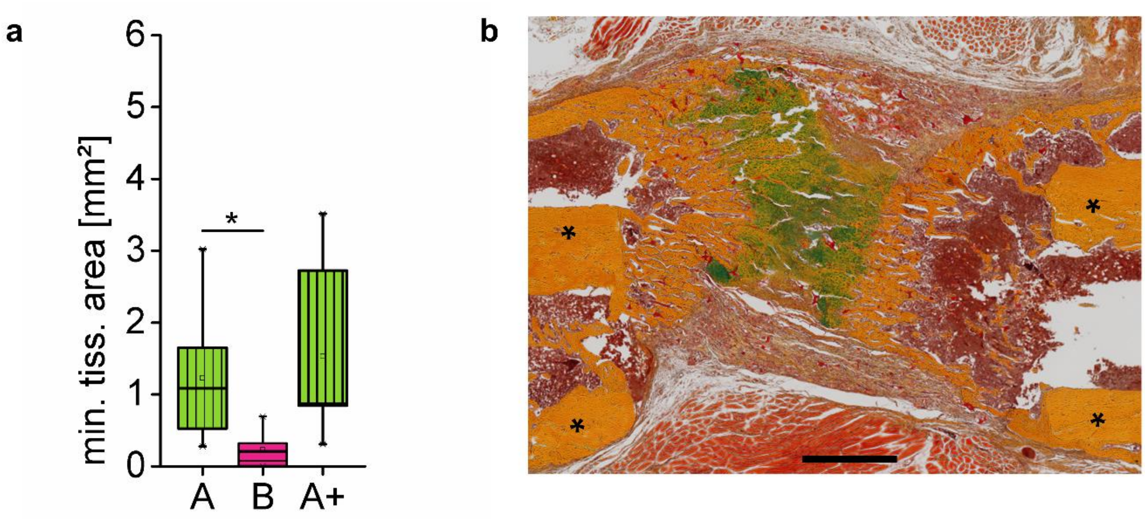

Supplementary Figure 11 Complementary analysis of bone healing using a scaffold A+ with increased mechanical stiffness (3% collagen, E=26.7(±2.8)kPa [mean±SD]) compared to scaffold A (1.5% collagen, E=8.5(±1.8)kPa [mean±SD]). **a** Histomorphometric evaluation of mineralized tissue area inside the scaffold shows that the stiffness of the scaffold has little effect on the bone healing capacity in contrast to the orientation of the scaffold pores (scaffold A vs. scaffold B). **b** Movat's pentachrome staining showing EO in scaffold A+ with cartilage (green) at the front of the mineralizing callus. Also the incidence of EO found 6 weeks post-op was comparable between scaffold A and A+ (3/8 animals with cartilage in scaffold A, 2/7 with cartilage in scaffold A+). Scale bar 1 mm.

<table>
<tr><th rowspan="2">Figure</th><th rowspan="2">groups compared</th><th rowspan="2">U</th><th rowspan="2">Z</th><th rowspan="2">asymp. probability>|U|</th><th rowspan="2">group</th><th rowspan="2">sample size (n)</th><th colspan="2">Bonferroni-correction</th><th>Effect size</th></tr>
<tr><th>p</th><th>p'</th><th>$\eta = \sqrt{\frac{Z^2}{n}}$</th></tr>
<tr><td colspan="10">Statistical test: Mann-Whitney test (two-sided) and Bonferroni correction</td></tr>
<tr><td>Fig. 5j</td><td>A vs. R; 3w</td><td>32.50</td><td>1.59</td><td>0.1111</td><td></td><td></td><td></td><td></td><td>0.4259</td></tr>
<tr><td></td><td>A vs. B; 3w</td><td>37.50</td><td>2.40</td><td>0.0162 *</td><td></td><td></td><td></td><td></td><td>0.6668</td></tr>
<tr><td></td><td>A vs. E; 3w</td><td>33.00</td><td>2.59</td><td>0.0096 *</td><td></td><td></td><td></td><td></td><td>0.7181</td></tr>
<tr><td></td><td></td><td></td><td></td><td></td><td>A</td><td>7</td><td></td><td></td><td></td></tr>
<tr><td></td><td></td><td></td><td></td><td></td><td>R</td><td>7</td><td></td><td></td><td></td></tr>
<tr><td></td><td></td><td></td><td></td><td></td><td>B</td><td>6</td><td></td><td></td><td></td></tr>
<tr><td></td><td></td><td></td><td></td><td></td><td>E</td><td>6</td><td></td><td></td><td></td></tr>
<tr><td></td><td></td><td></td><td></td><td></td><td></td><td></td><td>0.0500</td><td>0.0167</td><td></td></tr>
<tr><td></td><td></td><td></td><td></td><td></td><td></td><td></td><td>0.0100</td><td>0.0033</td><td></td></tr>
<tr><td></td><td></td><td></td><td></td><td></td><td></td><td></td><td>0.0010</td><td>0.0003</td><td></td></tr>
<tr><td></td><td>A vs. B; 6w</td><td>38.50</td><td>1.65</td><td>0.0980</td><td></td><td></td><td></td><td></td><td>0.4272</td></tr>
<tr><td></td><td>A vs. E; 6w</td><td>30.50</td><td>0.97</td><td>0.3319</td><td></td><td></td><td></td><td></td><td>0.2593</td></tr>
<tr><td></td><td>A vs. V; 6w</td><td>30.50</td><td>0.97</td><td>0.1660</td><td></td><td></td><td></td><td></td><td>0.2593</td></tr>
<tr><td></td><td></td><td></td><td></td><td></td><td>A</td><td>8</td><td></td><td></td><td></td></tr>
<tr><td></td><td></td><td></td><td></td><td></td><td>B</td><td>7</td><td></td><td></td><td></td></tr>
<tr><td></td><td></td><td></td><td></td><td></td><td>E</td><td>6</td><td></td><td></td><td></td></tr>
<tr><td></td><td></td><td></td><td></td><td></td><td>V</td><td>6</td><td></td><td></td><td></td></tr>
<tr><td></td><td></td><td></td><td></td><td></td><td></td><td></td><td>0.0500</td><td>0.0250</td><td></td></tr>
<tr><td></td><td></td><td></td><td></td><td></td><td></td><td></td><td>0.0100</td><td>0.0050</td><td></td></tr>
<tr><td></td><td></td><td></td><td></td><td></td><td></td><td></td><td>0.0010</td><td>0.0005</td><td></td></tr>
<tr><td colspan="10">Statistical test: Mann-Whitney test (two-sided) and Bonferroni correction</td></tr>
<tr><td>Fig. 5k</td><td>A vs. R; 3w</td><td>30</td><td>0.6389</td><td>0.5229</td><td></td><td></td><td></td><td></td><td>0.1707</td></tr>
<tr><td></td><td>A vs. B; 3w</td><td>32.00</td><td>1.50</td><td>0.1336</td><td></td><td></td><td></td><td></td><td>0.4160</td></tr>
<tr><td></td><td></td><td></td><td></td><td></td><td>A</td><td>7</td><td></td><td></td><td></td></tr>
<tr><td></td><td></td><td></td><td></td><td></td><td>R</td><td>7</td><td></td><td></td><td></td></tr>
<tr><td></td><td></td><td></td><td></td><td></td><td>B</td><td>6</td><td></td><td></td><td></td></tr>
<tr><td></td><td></td><td></td><td></td><td></td><td></td><td></td><td>0.0500</td><td>0.0250</td><td></td></tr>
<tr><td></td><td></td><td></td><td></td><td></td><td></td><td></td><td>0.0100</td><td>0.0050</td><td></td></tr>
<tr><td></td><td></td><td></td><td></td><td></td><td></td><td></td><td>0.0010</td><td>0.0005</td><td></td></tr>
<tr><td></td><td>A vs. B; 6w</td><td>51.00</td><td>2.60</td><td>0.0092 **</td><td></td><td></td><td></td><td></td><td>0.6723</td></tr>
<tr><td></td><td></td><td></td><td></td><td></td><td>A</td><td>8</td><td></td><td></td><td></td></tr>
<tr><td></td><td></td><td></td><td></td><td></td><td>B</td><td>7</td><td></td><td></td><td></td></tr>
</table>

Statistical test: Mann-Whitney test (two-sided) and Bonferroni correction									
**Fig. 6** (boxplot)	cartilage vs. no cartilage	299.00	4.82	**1.5E-06 ******					0.7921
					cartilage	12			
					no cartil.	25			

Statistical test: Mann-Whitney test (two-sided) and Bonferroni correction									
**Fig. 7b**	A vs. R; 3w	0.00	-2.93	**0.0034 ***					0.8122
	A vs. B; 3w	3.00	-2.32	0.0202					0.6703
	A vs. E; 3w	0	-2.647	**0.0081 ***					0.7982
					A	6			
					R	7			
					B	6			
					E	5			
							0.0500	0.0167	
							0.0100	0.0033	
							0.0010	0.0003	

Statistical test: Mann-Whitney test (two-sided) and Bonferroni correction									
**Fig. 7c**	A vs. R; 3w	35	2.6421	**0.0082 ***					0.7627
	A vs. B; 3w	34	2.482	**0.0131 ***					0.7165
	A vs. E; 3w	30	2.6473	**0.0081 ***					0.7982
					A	6			
					R	6			
					B	6			
					E	5			
							0.0500	0.0167	
							0.0100	0.0033	
							0.0010	0.0003	

Statistical test: Mann-Whitney test (two-sided) and Bonferroni correction									
**Fig. 8a**	A vs. R; 3w	76	1.9753	0.048					0.442
	A vs. B; 3w	33	-2.2016	0.028					0.449
	A vs. E; 3w	85	1.1386	0.255					0.237
					A	11			
					R	9			
					B	13			
					E	12			
							0.0500	0.0167	
							0.0100	0.0033	
							0.0010	0.0003	

Statistical test: Mann-Whitney test (two-sided) and Bonferroni correction									
**Fig. 8b**	A vs. R; 3w	39	-0.7597	0.447					0.1699
	A vs. B; 3w	4	-3.8818	**1.04 E-4 *** **					0.7924
	A vs. E; 3w	18	-2.9234	**0.0035 ***					0.6095
					A	11			
					R	9			
					B	13			
					E	12			
							0.0500	0.0167	
							0.0100	0.0033	
							0.0010	0.0003	
							0.0001	3 E-5	

Statistical test: Mann-Whitney test (two-sided) and Bonferroni correction									
**Fig. 8e**	A vs. B; 6w	58	2.678	**0.0037 ****					0.6695
	A vs. E; 6w	37	0.9837	0.1626					0.2540
					A	8			
					B	8			
					E	7			
							0.0500	0.0250	
							0.0100	0.0050	
							0.0010	0.0005	

Statistical test: Mann-Whitney test (two-sided) and Bonferroni correction									
**Fig. S3a**	A vs. R; 3w	32	1.5	0.1336					0.4160
	A vs. B; 3w	27	1.3611	0.1735					0.3929
	A vs. E; 3w	28	1.5212	0.1282					0.4391
					A 3w	6			
					R 3w	7			
					B 3w	6			
					E 3w	6			
							0.0500	0.0167	
							0.0100	0.0033	
							0.0010	0.0003	
	A vs. B; 6w	44	1.7938	0.0729					0.4632
	A vs. E; 6w	29	0.581	0.5613					0.1553
	A vs. V; 6w	42	2.2592	**0.0119 ***					0.6038
					A 6w	8			
					B 6w	7			
					E 6w	6			
					V 6w	6			
							0.0500	0.0167	
							0.0100	0.0033	
							0.0010	0.0003	

Statistical test: Mann-Whitney test (two-sided) and Bonferroni correction									
**Fig. S3b**	A 3w vs. A 6w	43	2.3883	**0.0085 ***					0.6383
	B 3w vs. B 6w	45	2.6465	**0.0041 ****					0.7073
					A 3w	6			
					A 6w	8			
					B 3w	6			
					B 6w	8			
							0.0500	0.0250	
							0.0100	0.0050	
							0.0010	0.0005	

Statistical test: Mann-Whitney test (two-sided) and Bonferroni correction									
**Fig. S3c**	A vs. V; 6w	46	0.1239	0.4507					0.0284
	A vs. B; 6w	56	0.0352	0.4860					0.0077
	A vs. E; 6w	58	-0.131	0.5522					0.0280
					A	11			
					V	8			
					B	10			
					E	11			
							0.0500	0.0167	
							0.0100	0.0033	
							0.0010	0.0003	

Statistical test: Mann-Whitney test (two-sided) and Bonferroni correction									
**Fig. S3d**	A vs. R; 3w	88	1.8133	0.0349					0.3866
non-min	A vs. B; 3w	64	1.0205	0.1537					0.2282
	A vs. E; 3w	31	-1.398	0.9190					0.3127
					A	10			
					R	12			
					B	10			
					E	10			
							0.0500	0.0167	
							0.0100	0.0033	
							0.0010	0.0003	
	A vs. R; 3w	88	2.3815	**0.0086 ***					0.5197
min	A vs. B; 3w	43	0.1766	0.4299					0.0416
	A vs. E; 3w	38	-0.531	0.7022					0.1218
					A	9			
					R	12			
					B	9			
					E	10			
							0.0500	0.0167	
							0.0100	0.0033	
							0.0010	0.0003	

Supplementary Table 1 Details of the statistical analysis performed. Bold numbers in column “asymp. probability” indicate statistical significance at the according level (* $p<0.05$, ** $p<0.01$, *** $p<0.001$,**** $p<0.0001$) taking into account Bonferroni correction for multiple comparisons . Higher n-numbers in statistics for Figures 6, 8a, 8b, S3c, S3d are resulting from individual analysis of proximal and distal callus in each animal.
